# Lifestyle-specific *S-*nitrosylation of protein cysteine thiols regulates *Escherichia coli* biofilm formation and resistance to oxidative stress

**DOI:** 10.1038/s41522-021-00203-w

**Published:** 2021-04-13

**Authors:** Nicolas Barraud, Sylvie Létoffé, Christophe Beloin, Joelle Vinh, Giovanni Chiappetta, Jean-Marc Ghigo

**Affiliations:** 1 Genetics of Biofilms Laboratory, Institut Pasteur, UMR CNRS2001, Paris, France; 2 Biological Mass Spectrometry and Proteomics (SMBP), ESPCI Paris, Université PSL, CNRS FRE2032, 75005, Paris, France

**Keywords:** Biofilms, Bacteriology

## Abstract

Communities of bacteria called biofilms are characterized by reduced diffusion, steep oxygen, and redox gradients and specific properties compared to individualized planktonic bacteria. In this study, we investigated whether signaling via nitrosylation of protein cysteine thiols (*S*-nitrosylation), regulating a wide range of functions in eukaryotes, could also specifically occur in biofilms and contribute to bacterial adaptation to this widespread lifestyle. We used a redox proteomic approach to compare cysteine *S-*nitrosylation in aerobic and anaerobic biofilm and planktonic *Escherichia coli* cultures and we identified proteins with biofilm-specific *S-*nitrosylation status. Using bacterial genetics and various phenotypic screens, we showed that impairing *S*-nitrosylation in proteins involved in redox homeostasis and amino acid synthesis such as OxyR, KatG, and GltD altered important biofilm properties, including motility, biofilm maturation, or resistance to oxidative stress. Our study therefore revealed that *S-*nitrosylation constitutes a physiological basis underlying functions critical for *E. coli* adaptation to the biofilm environment.

## Introduction

The formation of surface-attached communities of bacteria embedded in a matrix called biofilms provides a microenvironment preserved from external variations, which allows biofilms to colonize most surfaces, with both positive or negative ecological, medical and industrial consequences^1,2^. Compared to free-floating, individualized planktonic bacteria, biofilms develop specific metabolic capabilities, including a high tolerance to antimicrobials and host immune defenses^3–5^. However, whereas the understanding of bacterial adaptations to the biofilm lifestyle could provide clues for biofilm control, the physiological bases underlying these adaptations are still poorly understood.

One key aspect of the biofilm microenvironment is its physicochemical heterogeneity regarding levels of nutrient, wastes, or oxygen (O_2_)^6^. Indeed, steep O_2_ gradients were shown to develop rapidly in the three-dimensional biofilm structure^7–10^ and transcriptomics studies in facultative or strict aerobes such as *Escherichia coli* or *Pseudomonas aeruginosa* revealed gene expression profiles consistent with metabolic adaptations to biofilm microaerobic or anaerobic conditions^11–15^.

Changes in O_2_ levels in biofilms have important physiological consequences due to alteration of redox conditions and redox-mediated signaling in response to endogenous or exogenous oxidative or nitrosative stress^16–19^. Metabolic redox processes often generate reactive oxygen and nitrogen intermediates such as hydroxyl radical (OH·) leading to hydrogen peroxide (H_2_O_2_), or nitric oxide (NO) produced during anaerobic respiration on nitrate^20,21^. These highly reactive intermediates can, in turn, activate various signaling pathways via covalent binding to protein sensors including cysteine thiols, heme and nonheme metal centers, or iron–sulfur clusters^22–24^.

Cysteine thiols are of particular interest as they are involved in a range of reversible redox states from reduced S-H (oxidation number −2), disulfide bridges S-S (−1), nitrosylated S-NO (0), or sulfenic acids S-OH (0), associated with profound changes in corresponding protein functions^25^. However, whereas the formation of disulfide bridges in proteins exported to the periplasmic space or under conditions of oxidative stress is a well-studied process in bacteria^26^, much less is known about bacterial *S*-nitrosylation redox signaling. This reversible, enzymatically controlled modification involves the reaction of a nitrosonium cation NO+ with a reduced thiol^27^ and is known to regulate a wide range of cellular functions in plants and animals^28–30^, as well as host–microbe interactions, including pathogenesis and defense mechanisms^31–35^, or synergistic interactions^36^. However, redox sensors regulated by *S-*nitrosylation have been poorly studied in bacteria, except for the *E. coli* transcriptional regulator OxyR^37^. OxyR ability to bind target DNA sequences varies upon conformational changes that depend on its redox state, thus acting as a redox switch for OxyR-dependent transcription^38^. Previous studies showed that oxidative stress generated from H_2_O_2_ led to the formation of a disulfide bond between Cys199 and Cys208, activating an oxidative stress response in *E. coli*^39^. OxyR is also known to regulate other processes including adhesion and autoaggregation via phase variation regulation of the surface exposed autotransporter adhesin antigen 43, although the role of redox sensing in this signaling remains unclear^40–42^. Finally, recent works suggested that OxyR could adopt additional redox states, including sulfenic S-OH or nitrosylated S-NO, and regulate a specific set of genes under conditions of *S-*nitrosylation^43,44^.

In this study, we hypothesized that specific oxidation and nitrosylation patterns of cysteine thiols may occur in *E. coli* biofilm and not in planktonic conditions, thus contributing to the development of biofilm *S-*nitrosylation signaling and functions. We used a redox proteomics method combining the biotin-switch detection of protein *S*-nitrosylation with Stable Isotope Labeling in Cell Culture (SILAC) to identify proteins with biofilm-specific *S-*nitrosylated cysteine thiols. This approach showed that impairing *S*-nitrosylation status of proteins involved in redox homeostasis and amino acid synthesis affects *E. coli* biofilm formation and oxidative stress resistance, therefore identifying *S*-nitrosylation as a mechanism regulating functions critical for *E. coli* adaptation to the biofilm lifestyle.

## Results

### Development of a biotin-switch protocol to detect protein *S-*nitrosylation in *E. coli* planktonic and biofilm cultures

**Fig. 1:**
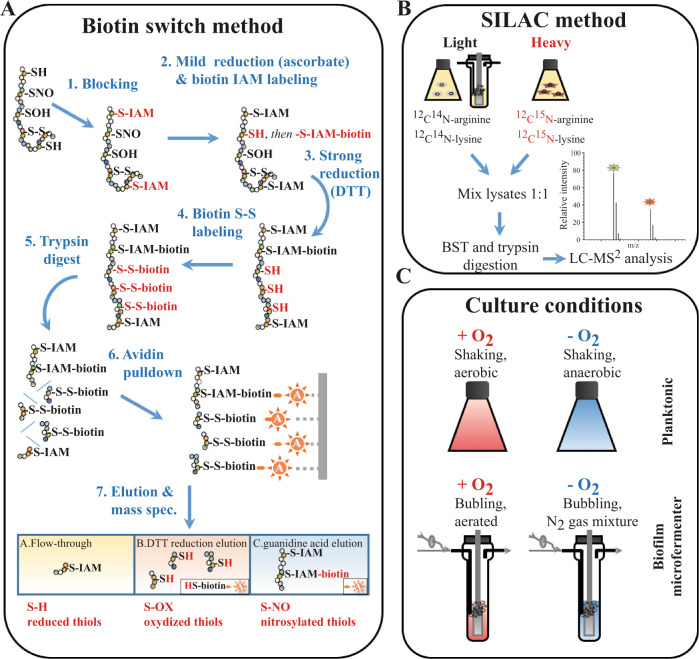
Workflow of the biotin-switch SILAC method used to identify and quantify redox-modified protein thiols in *E. coli* biofilm and planktonic conditions. **A** The biotin-switch method consists of the sequential blocking, reduction, and labeling of cysteine thiols; for the differential detection of S-NO and S-OX, cysteines are first reduced with ascorbate, a mild reducing agent, and then with the strong reducing agent DTT. Labeled peptides are then selectively eluted and collected for identification by mass spectrometry (LC-MS/MS). **B** Accurate peptide quantification using a SILAC approach was performed on each sample using heavy isotope lysine and arginine labeled samples as internal references. **C** *E. coli* proteins were extracted from four different cultures: planktonic shake flasks or biofilm microfermenters, each under aerobic or anaerobic conditions. Each condition included five biological replicates.

In order to detect *S-*nitrosylated (S-NO) peptides in *E. coli*, we adapted a previously described biotin-switch approach^45^ based on (1) the addition of iodoacetamide (IAM) to block free, reduced thiols, followed by (2) the mild and selective reduction of *S-*nitrosylated cysteines with ascorbate and IAM-biotin labeling (Fig. 1A). The extent of protein *S-*nitrosylation was then assessed by western blotting and immunodetection, taking advantage of avidin-biotin affinity with avidin-HRP antibodies.

**Fig. 2:**
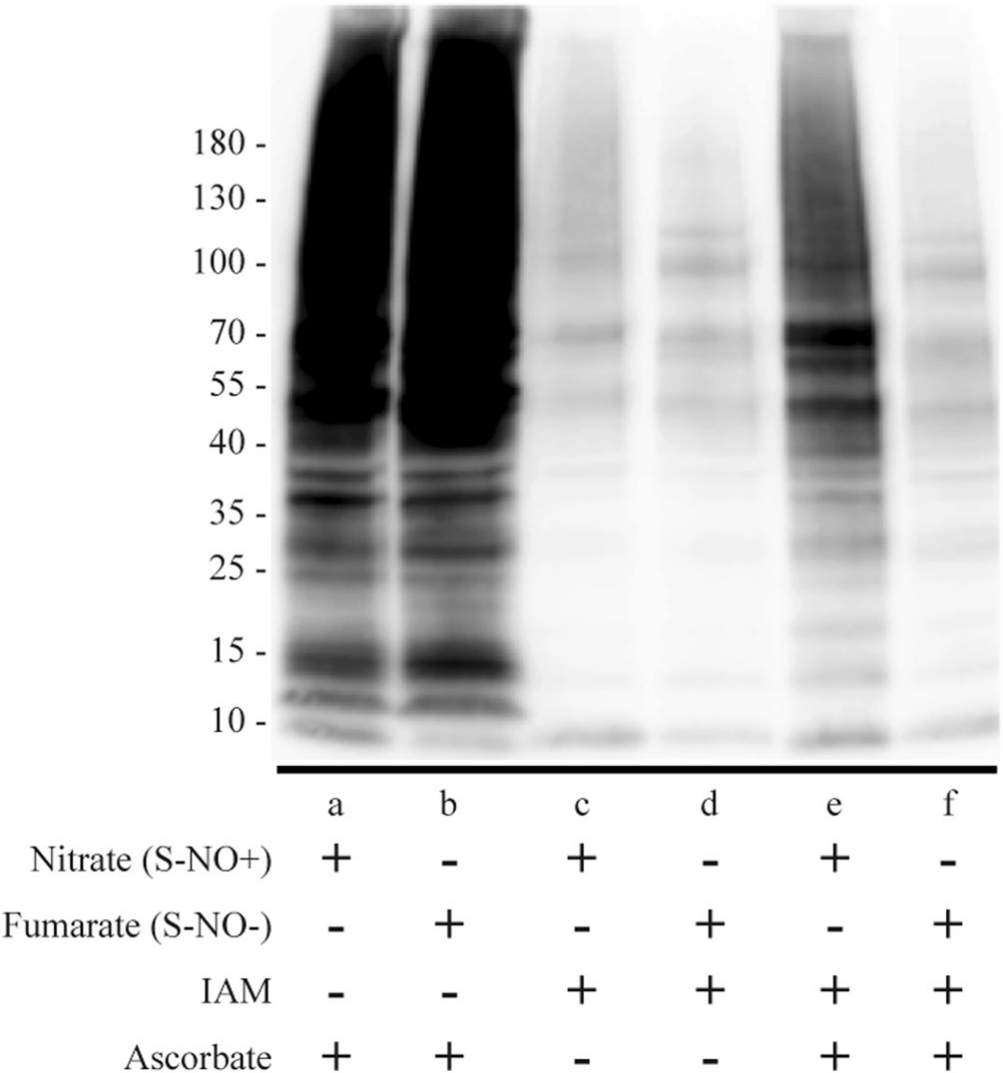
Validation of the biotin-switch method to detect protein *S-*nitrosylation in *E. coli*. Whole proteins were extracted from *E. coli* planktonic cultures grown under *S-*nitrosylating (S-NO+, anaerobic with excess nitrate), or non-*S-*nitrosylating conditions (S-NO−, anaerobic with fumarate). Proteins were then processed following the biotin-switch technique, except that some samples were not blocked with IAM (lanes a, b), or not reduced with ascorbate (lanes c, d), before labeling with IAM-biotin and immunodetection using avidin-HRP antibodies.

We validated the initial steps of the biotin-switch protocol using planktonic *E. coli* cultures grown in anaerobic conditions, either in the presence of excess amounts of nitrate, which leads to the accumulation of *S-*nitrosylated proteins (S-NO+ conditions), or in the presence of fumarate, preventing *S-*nitrosylation (S-NO− conditions)^43^. Total proteins extracted from 24 h cultures were processed following the biotin-switch protocol, except that some samples were either (1) not blocked with IAM, or (2) not reduced with ascorbate. In the absence of IAM, cysteines were biotinylated in all conditions (Fig. 2 lanes a, b). In contrast, the use of IAM blocking agent without subsequent reduction with ascorbate led to minimal biotin-labeled cysteine signal in both S-NO+ and S-NO− conditions (Fig. 2 lanes c, d), potentially corresponding to naturally biotinylated proteins. Finally, when protein cysteine thiols were blocked with IAM and S-NO specifically reduced with ascorbate, S-NO+ samples (nitrate conditions) showed abundant cysteine labeling (Fig. 2 lane e), while S-NO- samples (fumarate conditions) did not (Fig. 2 lane f). This indicated that the use of IAM and ascorbate could selectively label *E. coli S-*nitrosylated proteins in our experimental conditions.

**Fig. 3:**
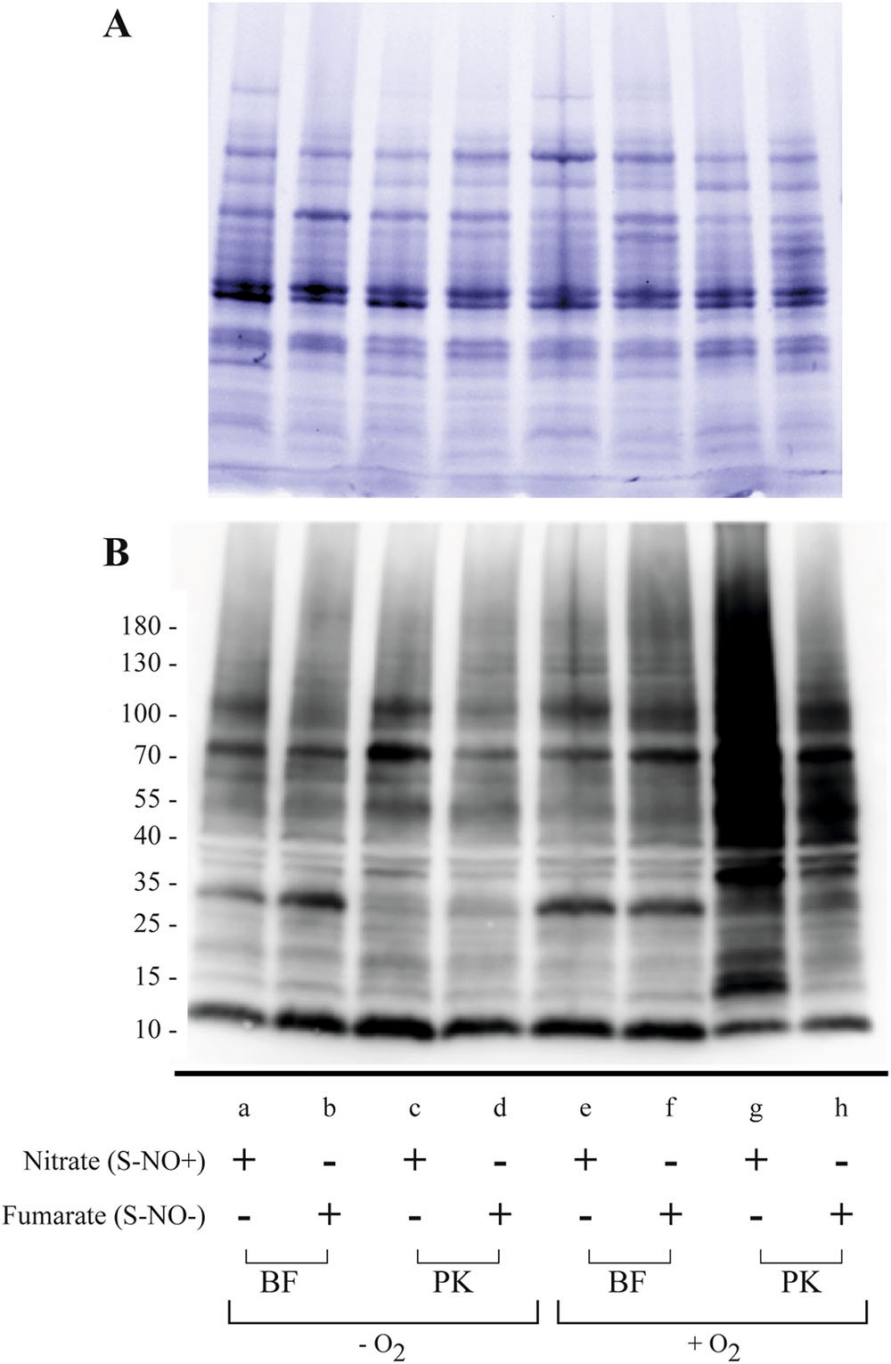
Protein *S-*nitrosylation profiles in *E. coli* planktonic (PK) or biofilm (BF) conditions. Cultures were grown under aerobiosis (+O_2_) or anaerobiosis (−O_2_) and under *S-*nitrosylating conditions with excess 10 mM nitrate (S-NO+), or non-*S-*nitrosylating condition supplemented with 10 mM fumarate (S-NO−). Proteins were extracted, processed with the biotin-switch method (see Fig. 1) and analyzed by western blot. **A** Total protein extracts visualized with Stain-free fluorescent detection; **B** S-NO proteins visualized by western blot followed by immunodetection using avidin-HRP antibodies.

To study potential modifications of protein *S-*nitrosylation profile in biofilms, we used continuous-flow biofilm microfermenters to grow mature *E. coli* biofilms in aerobic and anaerobic conditions in M63B1 glucose minimal medium supplemented with nitrate (S-NO+ conditions) or fumarate (S-NO− conditions) and we compared to the S-NO profiles of corresponding planktonic aerobic or anaerobic cultures (Fig. 1C). Biofilms grown in the absence of O_2_ and under conditions favoring *S-*nitrosylation (+nitrate) did not display more S-NO biotin-labeled signals compared to corresponding planktonic bacteria. However, biofilm S-NO labeled samples all displayed distinct band patterns, suggesting specific protein *S-*nitrosylation in biofilm bacteria (Fig. 3 compare lanes a and c). Furthermore, planktonic cultures showed increased *S-*nitrosylation when grown under aerobic compared to anaerobic conditions (Fig. 3 compare lanes c and g), whereas growing biofilm grown under high aeration did not drastically alter protein *S-*nitrosylation profile (Fig. 3 compare lanes a and e). Taken together, these results indicated that reduced availability of O_2_ availability in biofilms or anaerobic conditions led to reduced levels of *S-*nitrosylation.

### Redox proteomics analysis reveals fewer S-OX and S-NO cysteines but more reduced cysteines in biofilms compared to planktonic cultures

In order to identify proteins with cysteines specifically *S-*nitrosylated in biofilm and planktonic conditions, we combined our biotin-switch method with previously described redox proteomics workflow using SILAC (Fig. 1B)^45^. Each sample contained equal amounts of proteins originating from isogenic arginine and lysine *E. coli* auxotroph strains grown in the presence of the stable isotope-labeled (^13^C^15^N) L-arginine and L-lysine forms. In addition to the labeling of S-NO cysteines with IAM-biotin, reversibly oxidized cysteines (S-OX), including disulfide bridges and sulfenic acids, were labeled with biotin-HPDP. Hence, three peptide fractions were obtained after affinity enrichment: (1) the unbound (reduced cysteines and cysteine-free peptides), (2) the DTT fraction (S-OX), and (3) the guanidine fraction (S-NO) (Fig. 1A bottom).

The overall results of the redox proteomics analysis are summed up in Table 1, while the detailed datasets are reported in Supplementary Tables S1–S3. Volcano plot visualization of the data showed that the distributions of proteins and reduced cysteine fold changes were symmetric and overlapped between the two studied bacterial lifestyles (Supplementary Fig. S1). By contrast, the distributions of S-OX and S-NO cysteines deviated toward the planktonic mode of life, suggesting potential changes in cysteine site occupancy between the two bacterial lifestyles. According to site occupancy equations^46^, these trends suggested that the detected S-OX and S-NO cysteines involved a small fraction of the associated proteins.

**Table 1:** Summary of the redox proteomics results.

	Aerobic	Anaerobic	Uniques	Upregulated in PK conditions	Upregulated in BF conditions	
Protein	854	892	1426	93	99	
*S*-NO	161	132	198	21	6	Oxydation
*S*-Ox	357	299	409	33	11	
*S*-H	243	341	386	13	23	Reduction

Increases in S-NO and S-OX are considered as oxidation events, while increase in reduced fraction (SH) is considered as a reduction event.

Consistent with our preliminary immunodetection analysis (Fig. 3), we identified more cysteines significantly *S-*nitrosylated in planktonic than in biofilm bacteria (21 vs 6, see Tables 1–3). Our redox proteomics protocol also allowed us to detect S-OX cysteines (engaged in disulfide bonds or other reversible oxidation states), showing, similarly to S-NO cysteines, higher S-OX cysteine levels in planktonic conditions (Table 1, Supplementary Fig. S1, and Supplementary Tables S1 and S2). In agreement with this tendency, biofilms showed increased levels of reduced cysteine residues (Table 1, Supplementary Fig. S1, and Supplementary Tables S1 and S2). Overall, these data indicated a higher level of cysteine oxidation in planktonic conditions compared to biofilm cells.

**Table 2:** List of cysteine residues identified to be differentially *S*-nitrosylated in aerobically grown biofilm vs planktonic *E. coli* cultures by using the biotin-switch SILAC method.

Protein	Function	COG cat^a^	Cysteine position	Aerobic BFO_2_ vs PKO_2_
Biofilm-specific				
GldA	Glycerol dehydrogenase	C	C85	BF only
GltD^b^	Glutamate synthase (NADPH) small chain	E, R	C108	BF only
Planktonic-specific				
ArcA^b^	Aerobic respiration control protein	T, K	C173	PK only
PurC	Phosphoribosylaminoimidazole-succinocarboxamide synthase	F	C79	PK only
YtfE	Iron–sulfur cluster repair protein	O	C184	PK only
Aph	Aminoglycoside 3′-phosphotransferase	R	C31	PK only
GuaB	Inosine-5′-monophosphate dehydrogenase	R	C441	PK only
HisG	ATP phosphoribosyltransferase	E	C149	PK only
IleS	Isoleucine–tRNA ligase	J	C463	PK only
IleS	Isoleucine–tRNA ligase	J	C924	PK only
OxyR^b^	DNA-binding transcriptional dual regulator	K	C25	PK only
Pgl	6-phosphogluconolactonase	G	C152	PK only
PyrI	Aspartate carbamoyltransferase regulatory chain	F	C141	PK only
Rnr	Ribonuclease R	K	C118	PK only
RpsK	30S ribosomal protein S11	J	C70	PK only
LeuC	3-Isopropylmalate dehydratase large subunit	E	C75	−3.00
GlmS	Glutamine--fructose-6-phosphate aminotransferase	M	C2	−2.12
Ndk	Nucleoside diphosphate kinase	F	C139	−1.78
KatG^b^	Catalase hydroperoxidase I	P	C16	−1.75

Posttranslationally modified peptides that were detected only in one type of sample are listed as “BF only” (biofilm) or “PK only” (planktonic). Peptides with significant changes in S-NO profile with adjusted *p* values < 0.05 are shown (*n* = 5); data indicate fold changes in modified peptide normalized to total peptide count for biofilm compared to planktonic samples. Negative values indicate PKO_2_ vs BFO_2_ fold change.

*PKO*_*2*_ planktonic with O_2_, *BFO*_*2*_ biofilm with O_2_.

^a^COG categories^73^: C, energy production and conversion; D, cell cycle control, cell division, chromosome partitioning; E, amino acid metabolism and transport; F, nucleotide transport and metabolism; G, carbohydrate metabolism and transport; H, coenzyme transport and metabolism; J, translation, ribosomal structure, and biogenesis; K, transcription; L, cell wall/membrane/envelope biogenesis; M, cell wall/membrane/envelop biogenesis; O, posttranslational modification, protein turnover, chaperones; P, inorganic ion transport and metabolism; Q, secondary structure; R, general function prediction only; T, signal transduction; V, defense mechanisms.

^b^Proteins for which the *S*-nitrosylated cysteine was selected for cysteine-to-serine point mutation.

**Table 3:** List of cysteines identified to be differentially *S*-nitrosylated in anaerobically grown biofilm vs planktonic *E. coli* cultures by using the biotin-switch SILAC method.

Protein	Function	COG cat^a^	Cysteine position	Anaerobic BFn vs PKn
Biofilm-specific				
GrxC^b^	Glutaredoxin 3	O	C66	BF only
LeuD^b^	3-Isopropylmalate dehydratase small subunit	E	C82	BF only
LuxS^b^	S-ribosylhomocysteine lyase	T	C41	94.56
IlvE^b^	Branched-chain-amino acid aminotransferase	E, H	C42	38.42
Planktonic-specific				
AroG	3-Deoxy-7-phosphoheptulonate synthase	E	C208	PK only
LuxS^b^	S-Ribosylhomocysteine lyase	T	C128	PK only
RplJ	50S ribosomal protein L10	J	C71	PK only
OxyR^b^	DNA-binding transcriptional dual regulator	K	C25	PK only^c^
AtpD	ATP synthase subunit beta	C	C138	−6.66
KatG^b^	Catalase hydroperoxidase I	P	C16	−1.95

Posttranslationally modified peptides that were detected only in one type of sample are listed as “BF only” (biofilm) or “PK only” (planktonic). Peptides which showed significant changes in S-NO profile with adjusted *p* values < 0.05 are shown (*n* = 5); data indicate fold changes in modified peptide normalized to total peptide count for biofilm compared to planktonic samples. Negative values indicate PKn vs BFn fold change.

*PKn* planktonic without O_2_, *BFn* biofilm without O_2_.

^a^COG categories^73^ as in Table 1.

^b^Proteins for which the *S*-nitrosylated cysteine was selected for cysteine-to-serine point mutation.

^c^OxyR-C25-S-NO was detected in only three replicates in anaerobically grown planktonic cells and in none of the anaerobically grown biofilm samples (as indicated in “Materials and methods”, the selection threshold used in this study was four detections in one condition and none in the other).

### Lifestyle-specific *S-*nitrosylated proteins are involved in amino acid synthesis and redox homeostasis

Among the differentially *S*-nitrosylated proteins, the most represented functional category belonged to the COG E category, corresponding to amino acid metabolism and transport. Thus, several of the most highly *S*-nitrosylated peptides in biofilms were involved in amino acid synthesis including GltD under aerobic conditions and LeuD and IlvE under anaerobic conditions. By contrast, in planktonic bacteria, HisG, LeuC, and IleS were specifically *S-*nitrosylated in aerobic conditions, while AroG was *S*-nitrosylated in anaerobic conditions.

Several proteins involved in redox homeostasis control were differentially *S-*nitrosylated in biofilms compared to planktonic bacteria. Under anaerobic conditions, GrxC was more *S-*nitrosylated in biofilms (Table 2). Under aerobic conditions, ArcA, a regulator of aerobic respiration, and YtfE an iron–sulfur cluster repair protein recently found to be involved in nitrosative stress mediation^47^, were more *S-*nitrosylated in planktonic cells (Table 1). Furthermore, the catalase hydroperoxidase II KatG and the major dual redox regulator OxyR appeared to be *S-*nitrosylated in planktonic bacteria under both aerobic and anaerobic conditions. These results were consistent with varying oxidative conditions in biofilms and the associated presence of gradients of O_2_ or other electron acceptors. Surprisingly, the change in S-NO modification for OxyR was on Cys25, a thiol that had not been identified as *S-*nitrosylated in previous studies using planktonic *E. coli*^43,44,48^.

**Fig. 4:**
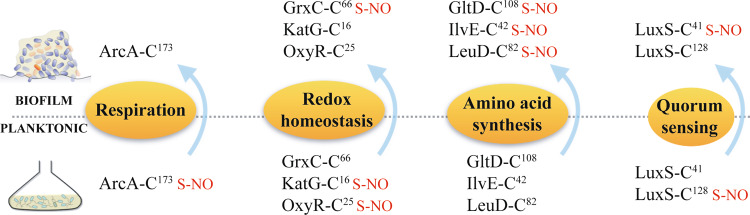
Cysteines with the highest *S-*nitrosylation fold changes or differentially *S-*nitrosylated in both aerobic and anaerobic conditions. These cysteines are in proteins involved in known biofilm-associated phenotypes, including respiration, redox homeostasis, amino acid synthesis, and quorum sensing.

Finally, LuxS, a central element of the quorum sensing cell–cell signaling system, showed a transfer of nitrosylation site from Cys128 in planktonic to Cys41 in biofilms under anaerobic conditions (Fig. 4).

### Impairing *S-*nitrosylation status of OxyR, KatG, and GltD affects biofilm-associated phenotypes

To investigate the phenotypic consequences of *S-*nitrosylation, we selected proteins displaying high cysteine *S-*nitrosylation fold changes in biofilm (GltD-Cys108, GrxC-Cys66, LeuD-Cys82, IlvE-Cys42, LuxS-Cys41) or planktonic conditions (ArcA-Cys173, LuxS-Cys128), or that were found to be S-nitrosylated in both anaerobic and aerobic planktonic conditions (OxyR-Cys25 and KatG-Cys16). These cysteines did not have lifestyle-associated modification of their oxidation or reduction status, suggesting a specific *S-*nitrosylation biofilm pattern (Supplementary Tables S1 and S2). We then introduced chromosomal and markerless point mutations changing the codon corresponding to the identified S-NO cysteine by a serine codon (C to S mutation). Cysteine to serine mutations in GrxC (C66S), LeuD (C82S), IlvE (C42S), LuxS (C41S and C128S), or ArcA (C173S) had no detectable impact on growth rate, biofilm formation, H_2_O_2_ sensitivity or motility (Supplementary Fig. S2). Moreover, we tested the potential impact of LuxS cysteine nitrosylation on AI-2 production using luminescent reporter *Vibrio harveyi* strains responsive to AI-2. We observed that luminescent signals induced by the presence of AI-2 from the supernatant of *E. coli* MG1655 *luxS*_C41S_ or *luxS*_C128S_ are similar to the one induced by WT supernatant, indicating that the nitrosylation status of Cys41 and Cys128 does not influence AI-2 production and therefore is not likely to influence the expression of AI-2 controlled genes (Supplementary Fig. S3).

**Fig. 5:**
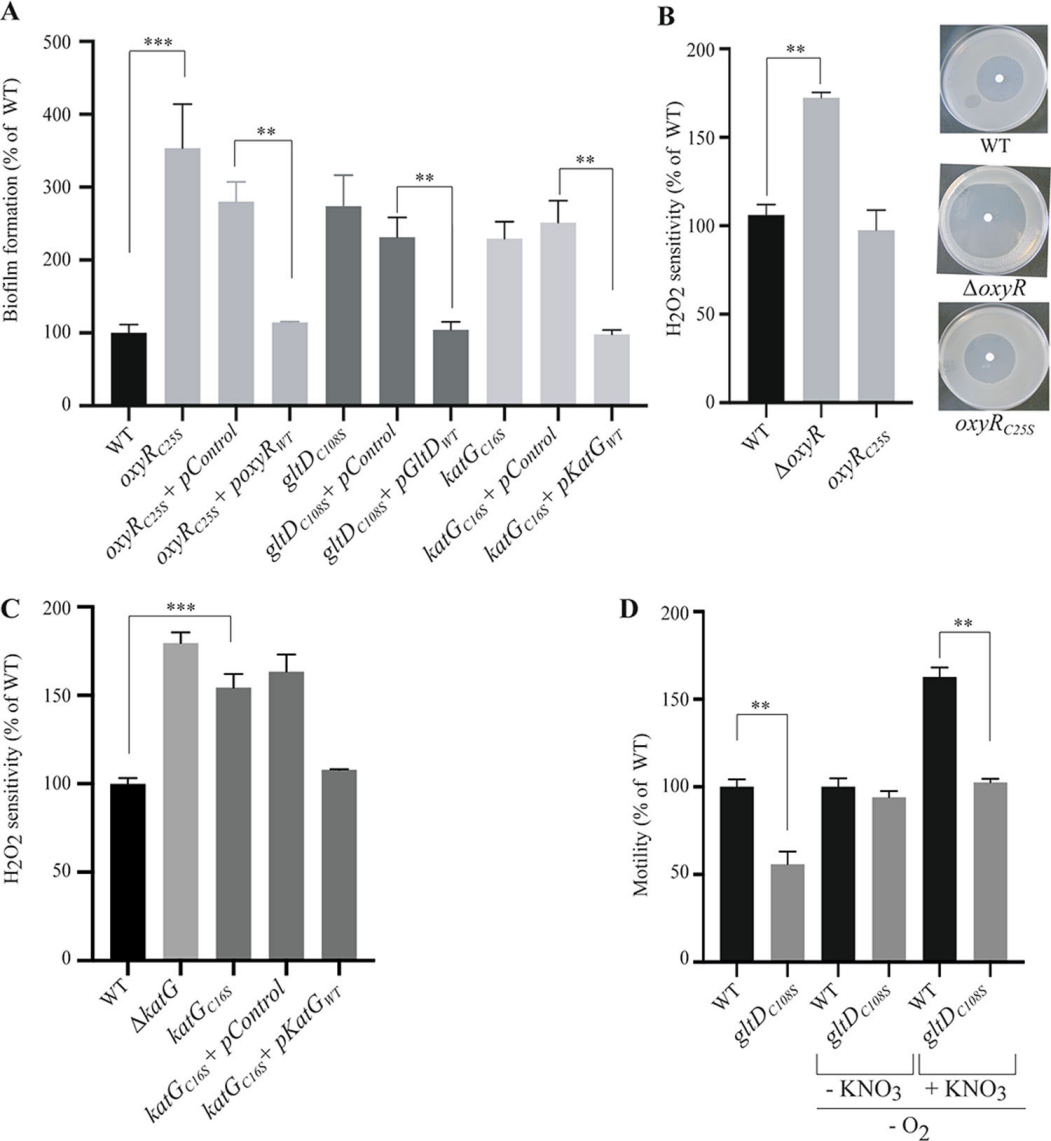
Impairing *S*-nitrosylation status of OxyR, KatG, and GltD affects biofilm-associated phenotypes. **A** Biofilms of *E. coli* WT, *oxyR*_C25S,_ *katG*_C16S_, and *gltD*_C108S_ mutants, complemented or not with the corresponding plasmid-based allele or empty vector, were grown in continuous-flow microfermenters for 24 h in LB medium before quantifying the biofilm biomass. The level of biofilm formed by the WT strain was set to 100%. **B** Sensitivity to H_2_O_2_ oxidative stress of an *oxyR*_C25S_ mutant compared to the WT and ∆*oxyR* mutant. The distance of growth inhibition from the edge of the disk to the edge of the growth zone was measured and was set to 100% for the WT strain. **C** Increased sensitivity to oxidative stress of *katG*_C16S_ mutant. The sensitivities to H_2_O_2_ of *E. coli* WT and *katG*_C16S_ mutant complemented with corresponding plasmid-based allele or empty vector were compared. The distance from the edge of the disk to the edge of the growth zone was measured and was set to 100% for the WT strain. **D** *S*-nitrosylation-dependent decreased motility of *gltD*_C108S_ mutant. The motility of *E. coli* WT and *gltD*_C108S_ mutant were compared in aerobic conditions and in anaerobic conditions in presence of KNO_3_. Assays were performed on 0.3% agar plates and incubated overnight at 30 °C. All experiments were performed in triplicate, mean values are reported and error bars represent standard deviations. **p ≤ 0.05, ***p ≤ 0.01. See also Figs. S2, S5 and S6.

We also showed that a mutant in the hybrid cluster protein Hcp previously found to regulate protein *S*-nitrosylation and mediate bacterial motility under conditions of *S*-nitrosylation has no impact on biofilm formation (Supplementary Fig. S4)^44^. By contrast, *oxyR*_C25S_, *katG*_C16S_, and *gltD*_C108S_ mutants displayed growth rates similar to the WT (Supplementary Fig. S2), but showed increased biofilm formation compared to WT, a phenotype observed both in rich (Fig. 5A) and minimal medium (Supplementary Fig. S5), and which could be complemented by introducing the corresponding plasmid-based WT allele of *oxyR*, *katG*, or *gltD* into the corresponding mutant strains (Fig. 5A).

The cysteines differentially *S*-nitrosylated in biofilms in OxyR, KatG, or GltD did not show any biofilm-specific reversible oxidation or reduction (Supplementary Tables S1 and S2), except for OxyR-Cys25, which was more reduced in anaerobic planktonic conditions. This suggested that the altered phenotypes associated with the C to S mutations were due to altered *S*-nitrosylation rather than due to another modification of the redox status of these thiols. The strain carrying the *oxyR*_C25S_ mutation also showed increased cell aggregation, similar to the one of a ∆*oxyR* strain, in which phase variable expression of Ag43 autotransporter self-aggregation adhesin is locked ON (Supplementary Fig. S6A)^49^. Consistently, *E. coli oxyR*_C25S_ biofilm phenotype depended on the presence of *flu*, the gene encoding Ag43 adhesin (Supplementary Fig. S6B), displayed 100% ON colonies (Supplementary Fig. S6C), and an increased level of Ag43 protein, similarly to a *∆oxyR* mutant (Supplementary Fig. S6D). Although this suggested that preventing Cys25 *S-*nitrosylation phenocopied an *oxyR* deletion, we did not observe the characteristic increased sensitivity to H_2_O_2_ oxidative stress, indicating that preventing OxyR *S-*nitrosylation affected some but not all OxyR functions (Fig. 5B). To assess the specificity of C25S mutation, we also tested a Q29S mutant potentially impairing OxyR DNA-binding site and found that this strain had similar phenotypes as the C25S mutant: increased biofilm formation and no impact on H_2_O_2_ sensitivity (Supplementary Fig. S7). In contrast to *oxyR*_*C*25S_, the *katG*_C16S_ mutant exhibited increased sensitivity to oxidative stress upon exposure to H_2_O_2_ compared to WT (Fig. 5C and Supplementary Fig. S5), showing that planktonic *S-*nitrosylation could protect against oxidative stress. On the other hand, whereas the *gltD*_C108S_ mutation had no impact on sensitivity to oxidative stress (Supplementary Fig. S2), its increased biofilm formation capacity corresponded with a reduced motility phenotype in aerobic condition and in anaerobic condition with KNO_3_ (S-NO+ conditions), indicating that GltD-Cys108 *S-*nitrosylation increases motility in biofilm bacteria (Fig. 5D and Supplementary Fig. S5). Taken together, these results showed that altering *S*-nitrosylation can trigger an array of functions relevant to the switch between the biofilm and planktonic lifestyles.

## Discussion

Bacterial biofilms are characterized by steep O_2_ gradients leading to microaerobic or anaerobic zones, profoundly affecting the physiology of biofilm bacteria. In this study, we used redox proteomics and bacterial genetic and phenotypic analyses to identify nitrosylated cysteines altering biofilm functions.

The addition of a reduction and labeling step to a previously described *S-*oxidized (S-OX) cysteine biotin-switch protocol^45^, combined with SILAC allowed us to selectively detect and quantify *S-*nitrosylated (S-NO) cysteines and other redox protein modifications in *E. coli* biofilm and planktonic bacteria. We showed that proteins extracted from planktonic bacteria possess more S-NO and S-OX cysteines compared to protein extracted from poorly oxygenated biofilm environment. Furthermore, increased *S-*nitrosylation of proteins in the presence of O_2_ was proposed to increase likelihood of NO moieties binding to cysteine thiols^50^, which is consistent with higher S-NO and S-OX levels detected in aerobiosis. Our approach allowed us to monitor global changes in protein expression profiles, comparing biofilms and planktonic cultures. Consistently with previous standard proteomic analyses^51^, the most downregulated proteins in aerobic biofilms corresponded to siderophore biosynthesis and iron transport (Supplementary Fig. S8). In contrast, upregulated proteins in aerobic biofilms were clustered into respiration, carbohydrate uptake, or amino acids biosynthesis functional class (Supplementary Fig. S9).

Our study showed that reversible redox modifications of proteins previously associated with the biofilm lifestyle, including redox homeostasis, amino acid synthesis, or respiration, occur during biofilm development. While these protein modifications may be a consequence of the biofilm reducing environment, the presence of biofilm-specific S-NO proteins also suggest that *S-*nitrosylation-dependent regulation could control biofilm functions. Recently, a multiplex enzymatic mechanism was identified for the regulation of cell motility and metabolism in *E. coli*, involving the hybrid cluster protein Hcp and nitrate reductase NarG. Under anaerobic conditions, Hcp was found to interact with several proteins to induce S-NO from NarG-derived NO as well as propagate S-NO-based signaling via trans-nitrosylation of proteins^44^. However, when we tested a Δ*hcp* mutant, no impact on biofilm formation was observed, suggesting that Hcp is not involved in the regulation of S-NO dependent biofilm formation.

Protein *S-*nitrosylation is known to regulate a wide range of critical physiological functions in eukaryotes^29,52^. Here, we demonstrated that disabling *S-*nitrosylation sites in OxyR, KatG, or GltD increases biofilm development. Since OxyR-Cys25 and KatG-Cys16 cysteines are *S*-nitrosylated in planktonic conditions under both aerobic and anaerobic conditions, this suggests that OxyR and KatG *S-*nitrosylation are associated with the switch from biofilm to planktonic lifestyle, either by directly inhibiting bacterial adhesion mechanisms and promoting biofilm dispersal or via an intermediate regulator. Furthermore, the increased sensitivity of *katG*_C16S_ mutant toward oxidative stress also indicates a role for KatG-Cys16 *S-*nitrosylation in the regulation of oxidative and nitrosative stress in *E. coli*^53^. In contrast to KatG, GltD-Cys108 was found to be *S*-nitrosylated in biofilm conditions. Taken together these results indicate that both denitrosylation (e.g. OxyR, KatG) and nitrosylation (e.g. GltD) mechanisms may be involved in biofilm formation. *S*-nitrosylation also appears to be linked to the regulation of oxidative stress response as impairing *S*-nitrosylation of KatG, in a *katG*_C16S_ mutant, led to increased sensitivity toward oxidative stress. This suggests that under conditions of physiological *S*-nitrosylation, here in planktonic cells compared to biofilm cells, the response to oxidative stress is reduced, while in contrast biofilm cells would maintain a fully active H_2_O_2_ protection machinery. Interestingly KatG and OxyR which are both S-nitrosylated in planktonic cells are directly related as OxyR positively regulates *katG* transcription under oxidative conditions^39^. However, because the *oxyR*_C25S_ mutant was not affected in this response, it is unlikely that OxyR-C25S *S*-nitrosylation regulates *katG* transcription, suggesting that cysteine nitrosylation plays a role in the oxidative stress response only via direct modulation of catalase activity.

OxyR is a well-known regulator of the phase variable switch of the autotransporter adhesin Ag43, a major *E. coli* surface protein involved in bacterial aggregation and biofilm formation. OxyR contains six cysteines, and Cys25 and Cys199 are the only solvent-accessible sites in the native protein^48^. Previous investigation of NO binding to OxyR cysteines by exposing His-tag purified OxyR to 20-fold molar excess of the NO donor S-nitrosoglutathione (GSNO)^43^ showed that Cys199 was predominantly *S-*nitrosylated in presence of 10 mM nitrate. However, the authors reported that Cys25 also showed low 20% S-NO signal, which they attributed to incomplete thiol blocking during a preliminary step^43^. Here we found that OxyR is *S-*nitrosylated on Cys25 in planktonic bacteria in the presence of 300 µM KNO_3_, which represents physiologically relevant levels of nitrate^54,55^, suggesting that Cys25 is a bona fide OxyR *S-*nitrosylation site. Furthermore, inactivating this S-NO site led to increased biofilm formation, similar to a *∆oxyR* mutant. By contrast, OxyR-Cys25 denitrosylation had no impact on *E. coli* sensitivity to oxidative stress, which is typically increased in the absence of OxyR by relieving inhibition of *oxyS* transcription^38,56^. The lack of effect of OxyR-Cys25 on oxidative stress correlates with previous studies showing that cysteine-to-serine mutation of Cys25 did not affect binding to the *oxyS* promoter region^57^. Cys25 is located in the OxyR DNA-binding site. Previous studies excluded the involvement of the Cys25 residue in OxyR dimerization, which was shown to require the formation of a disulfide bond between Cys199 and Cys208 as well as residues E126, R228, E248, H125, H218, M230, and S235 located at OxyR interaction interface^58–60^. The increased expression of Ag43 and bacterial aggregation and biofilm formation could be a consequence of the altered OxyR DNA-binding site in the *oxyR*_C25S_ mutant. However, previous studies showed that cysteine-to-serine mutation of Cys25 did not affect binding to the *oxyS* promoter region^57^. Moreover, an *oxyR*_Q29S_ mutant, potentially impairing OxyR DNA-binding site, also increased biofilm formation and had no impact on H_2_O_2_ sensitivity, which is consistent with the observed lack of effect of this mutation on resistance to H_2_O_2_. While these observations do not exclude that NO binding to Cys25 could affect Ag43 gene transcription, we cannot distinguish between phenotypes associated to removal of *S-*nitrosylation and impairment of DNA binding.

Overall this study revealed that proteins involved in functions broadly associated to biofilm physiology across various bacterial species are specifically *S*-nitrosylated in biofilms. The role played by *S*-nitrosylation in the regulation of biofilm development suggests that effectors of S-NO proteins could constitute new targets for biofilm control strategies. How these mechanisms are coordinated in time and space during biofilm development and whether specific enzymes such as nitrosylases or denitrosylases are involved remain to be elucidated.

## Methods

### Bacterial strains and culture media

Bacterial strains used in this study are listed in Supplementary Table S4. Bacterial planktonic cultures were grown in Lysogeny broth (LB) containing 1% (w/v) tryptone, 0.5% (w/v) yeast extract, and 1% (w/v) NaCl at 37 °C, supplemented with appropriate antibiotics when needed (kanamycin 50 µg/ml, carbenicillin 100 mg/ml, tetracycline 7.5 µg/ml). For biofilm and planktonic comparisons, cultures were grown in minimal medium M63B1 containing 100 mM KH_2_PO_4_, 15 mM (NH_4_)_2_SO_4_, 0.4 mM MgSO_4_, 10 µM FeSO_4_, 3 µM vitamin B1, pH 7.0, and supplemented with 0.1% (w/v) (biofilm) or 0.4% (planktonic) glucose (22 mM). To obtain proteins that were analyzed by mass spectrometry (SILAC), all biofilm and planktonic cultures were supplemented with 300 µM KNO_3_, which represents physiologically relevant levels of nitrate^54,55^. In contrast, for western blot analysis (biotin-switch method confirmation), biofilm and planktonic cultures were grown in the presence of 10 mM KNO_3_ or fumarate (excess amounts), as described previously to generate *S-*nitrosylation profiles detectable with this analysis^43^. For redox proteomics experiments, planktonic and biofilm cultures of auxotroph mutants were supplemented with 500 µM of L-arginine and L-lysine; or for the reference samples, planktonic cultures were supplemented with 500 µM of stable isotopes ^13^C_6_^15^N_4_ L-arginine and ^13^C_6_^15^N_2_ L-lysine (Pierce). All media and chemicals were purchased from Sigma-Aldrich or from specific suppliers as indicated.

### Single nucleotide mutant construction

Single nucleotide mutations were introduced in MG1655 F’*tet* genome using transient mutator multiplex automated genome engineering(MAGE)^61^. Primers consisting of 90 bp long ssDNA fragments harboring the targeted nucleotide mismatch and phosphorothioate bonds at the 5′ end were designed using the online MODEST tool available at http://modest.biosustain.dtu.dk^62^ and ordered from Sigma (see Table S5). First, λ-Red- and *dam*-containing plasmid pMA7SacB^63^ was transformed into *E. coli*. pMA7SacB-containing *E. coli* induced with 0.2% (w/v) L-arabinose and made electrocompetent by rinsing several times in cold deionized water were repeatedly transformed with 10 pmol of the specific primer^64^, and immediately regrown in LB with tetracycline and carbenicillin antibiotics. After 3–4 cycles, single clones were isolated and screened by PCR using a high discrimination HiDi polymerase (myPols) and primers with the targeted original or mutated nucleotide at the 3′ end (see Table S5). Finally, positive clones were checked by PCR with specific primers and DNA sequencing.

### Transduction of chromosomal mutation

Single nucleotide mutations were moved into naïve WT (MG1655 F’*tet*) background by introducing for each of these three strains, a kanamycin resistance marker immediately downstream of the mutated *oxyR*, *katG*, and *gltD* genes. We then performed a P1 transduction of the kanamycin marker into a WT *E. coli* background and checked that, owing to their genetic proximity each corresponding cysteine-to-serine mutation were co-transduced along with the kanamycin marker. *E. coli* gene deletion used in this study originated either from the *E. coli* Keio collection of mutants^65^ or were generated by λ-red linear DNA gene inactivation using pKOBEG or pKOBEGA plasmids^66^. Primers used to construct recombinogenic DNA fragments are listed in Supplementary Table S5. P1*vir* transduction was used to transfer mutations between different strains. When required, antibiotic resistance markers flanked by two FRT sites were removed using Flp recombinase^67^. Plasmids used in this study were constructed using an isothermal assembly method, Gibson assembly (New England Biolabs, Ipswich, MA, USA), using primers listed in Supplementary Table S5. The integrity of all cloned fragments, mutations, and plasmids was verified by PCR with specific primers and DNA sequencing.

### Construction and characterization of *flu-lacZ* transcriptional fusions

Deletion of chromosomal *lacZ* gene mutation (*∆lacIZ::Cm*) and a *endflu-lacZzeo* construct corresponding to a translational fusion between the *flu* gene encoding Ag43 autotransporter adhesin and the reporter gene *lacZ* were successively introduced by P1*vir* phage transduction in *E. coli* WT, ∆*oxyR::Km*, and *oxyR*_C25S_ strains. The ON (blue) or OFF status of Agn43 in these strains were assessed by resuspending colonies in 1 ml LB medium and plating dilutions on LB agar plates supplemented with 100 µg/ml of 5-bromo-4-chloro-3-indolyl-ß-D-galactopyranoside (X-gal). The plates were incubated overnight at 37 °C.

### Determination of AI-2 activity

Relative levels of AI-2 in bacterial-free culture supernatants of WT, *luxS*_C41S_, and *luxS*_C128S_ strains, in presence or absence of KNO_3_, were measured by using a *V. harveyi* bioluminescence assay, as described previously^68,69^. Briefly, *E. coli* WT, *luxS*_C41S_, and *luxS*_C128S_ strains were grown overnight with agitation in LB broth at 30 °C in presence or absence of 10 mM KNO_3_. Similarly, the control *V. harveyi* strain BB120 (AI-1^+^, AI-2^+^) was grown in AB medium overnight at 30 °C with aeration. Bacterial cells were removed by centrifugation and the resulting supernatants were subsequently filter sterilized through a 0.22-µm-pore size filter (Millipore). The luminescent reporter strain *V. harveyi* BB170 (sensor1^−^, sensor 2^+^) was grown for 18 h at 30 °C with aeration in AB medium and diluted 1:5000 into fresh AB medium. The cell-free supernatants (20 µl) were added to the diluted BB170 cells (180 µl) at a 10% (v/v) final concentration in hemolysis tubes, which were then shaken at 30 °C for 3 h. Positive and negative control samples were obtained by adding cell-free supernatant from *V. harveyi* BB120 and LB medium, respectively, to a final concentration of 10%. After the incubation period, the resulting light production of each sample was measured using a Tecan Infinite-M200-Pro spectrophotometer. AI-2 activity was expressed as luminescence relative units.

### Ag43 immunodetection

For each biofilm culture, the equivalent of 0.2 OD_600_ units was analyzed by sodium dodecyl sulfate-10%polyacrylamide gel electrophoresis, followed by immunodetection of Ag43 using a polyclonal rabbit antiserum raised against the a-domain of Ag43 at a dilution of 1:10,000. Protein loading accuracy was verified using staining of membrane with Ponceau S red.

### Biofilm and planktonic culture conditions

Biofilms were cultivated in continuous-flow microfermenters containing a removable glass spatula^70^ (see also https://research.pasteur.fr/en/tool/biofilm-microfermenters/). Sterile microfermenters were inoculated with 10^8^ bacteria from an overnight culture, and cells were allowed to attach for 1 h static at 37 °C before turning on the medium flow. Microfermenters were operated with a medium flow rate of 40 ml/h (residence time 40 min) and internal bubbling of a filter-sterilized compressed gas at ~0.2 bar_g_ consisting of: (1) air for aerobic conditions, or (2) a mix of 90% nitrogen, 5% hydrogen, 5% carbon dioxide (Air Liquide) for anaerobic conditions. Biofilm cultures were grown for 48 h before protein extraction. Planktonic cultures were inoculated to an optical density of the culture at 600 nm (OD_600_) of 0.005 and grown in Erlenmeier glass flasks at 37 °C, either: (1) in an aerated shaker incubator for aerobic conditions, or (2) in a Concept 400 M anaerobic workstation (Ruskinn) on a multi-position magnetic stirrer (Carl Roth) for anaerobic conditions. Planktonic cultures were grown for 24 h before protein extraction.

### Motility assay

Overnight cultures of the tested bacterial strains were spotted as a 2-µL drop of a 10^−2^ dilution onto 0.3% agar plates with 10 g/l Bacto tryptone, 5 g/l NaCl and 3 g/l agar, or with 3 g/l agar in M63B1 minimal medium. Motility plates were incubated overnight at 30 °C. The distance from the edge of the inoculation spot to the edge of the growth zone was measured.

### Oxidative stress assay

Overnight cultures in LB medium were diluted to an OD_600_ of 0.05 in fresh medium and then allowed to grow to an OD_600_ of 0.1. Then, 100 µl of each culture were spread on LB or M63B1 plates. Round sterile filters were placed in the center of the plates and spotted with 25 µl of 30% H_2_O_2_. Plates were incubated at 37 °C overnight. The distance from the edge of the disk to the edge of the growth zone was measured.

### Autoaggregation assay

Isolated colonies were used to inoculate 5 ml LB medium and grown overnight (16–18 h). The OD_600_ was adjusted to 3.0 by resuspension of the cell pellet in nutrient-exhausted LB medium (supernatant obtained from respective overnight grown cultures after centrifugation), and 3 ml of each adjusted culture were transferred to 5 ml hemolysis tubes. These tubes were incubated without agitation at room temperature and the OD_600_ of the upper part of each standing tube culture was determined every hour for 8 h.

### *S-*nitrosylated and oxidized thiols biotin-switch SILAC method

#### Protein extraction

*S*-oxidized and *S*-nitrosylated peptides were analyzed by applying a cysteine biotin-switch technique, as described before^45^, with some modifications. First, biofilm or planktonic bacteria were lysed with trichloroacetic acid (TCA) immediately after cultivation: biofilm cells on the fermenter spatula were first resuspended in cold phosphate-buffered saline (PBS) before centrifugation to obtain the cell pellet, while 25 ml of planktonic cells were immediately centrifuged for 10 min at 8000 × *g*. The cell pellet was resuspended in 0.6 ml of 20% (v/v) TCA, thus representing ~1.5–2.0 mg/ml protein content, and centrifuged at 4 °C, 16,000 × *g* for 1 h, before washing three times with ice-cold acetone.

#### R-SH blocking

Following extraction, reduced thiols were blocked by resuspending the pellet containing proteins and cell debris in HENS lysis buffer, consisting of 250 mM 4-(2-hydroxyethyl)-1-piperazineethanesulfonic acid, pH 7.7, 1 mM ethylenediaminetetraacetic acid, 0.1 mM neocuproine, 6 M urea, 1% (w/v) N-octyl-β-D-glucopyranoside, a protease inhibitor cocktail, and supplemented with 200 mM IAM, allowing for improved saturation of reduced thiols as shown in a previous study^71^. Protein content was adjusted to 0.8 mg/ml in a volume of 400 µl and the mixture was incubated at 37 °C with shaking at 2000 rpm for 1 h in the dark in a Thermomixer (Eppendorf).

For mass spectrometry analysis, each sample was mixed with one volume of a heavy isotope-labeled reference sample after this blocking step and further processed as one sample. The reference sample consisted of a 5:5:1 mixture of planktonic extracts corresponding to non-*S-*nitrosylating (aerobic with fumarate), mildly *S-*nitrosylating (anaerobic with 300 µM nitrate) and strongly *S-*nitrosylating (anaerobic with 10 mM nitrate) conditions.

#### R-SNO ascorbate reduction and IAM-biotin labeling

Proteins were precipitated with TCA and acetone before being resuspended in HENS buffer with 10 mM ascorbate and 2 mM EZ-Link iodoacetyl-PEG2-biotin (IAM-biotin, Thermo Scientific), and incubation at 37 °C with shaking at 2000 rpm for 1 h.

At this stage, proteins were either directly analyzed by western blot or further processed for R-S-OX reduction and labeling, peptide digestion, avidin pull-down and finally identification by mass spectrometry.

#### Western blot analysis

Proteins were precipitated with TCA and acetone before being resuspended in 300 µl 50 mM ammonium bicarbonate, at 37 °C with shaking at 2000 rpm for 1 h. Then the solution was mixed with 2X Laemmli buffer without reducing agent and loaded onto SDS-PAGE, transferred to cellulose membrane by using a Trans-Blot Turbo Transfer System (Bio-Rad), labeled with avidin-HRP conjugates (eBioscience) (1:10,000 dilution in PBS with 0.05% Tween), revealed with ECL Prime chemiluminescence reagents (GE Healthcare) and visualized in a G-Box (Syngene). Total proteins were visualized from SDS-PAGE gels with Stain-free fluorescent imaging (Bio-Rad).

#### R-SOX DTT reduction and S-S-biotin labeling

Proteins were precipitated with TCA and acetone before being resuspended in HENS buffer with 10 mM 1,4-dithiothreitol (DTT) and 0.5 mM EZ-Link HPDP-biotin (S-S-biotin, Thermo Scientific), and incubation at 60 °C with shaking at 2000 rpm for 30 min.

#### Trypsin digestion

Proteins were precipitated with TCA and acetone before being resuspended in 300 µl of 50 mM ammonium bicarbonate and digested with 1 µg/ml trypsin (Roche) at 37 °C, 1000 rpm overnight.

#### Avidin pull-down

One hundred microliters of streptavidin resin (Pierce) were loaded onto spin columns (Pierce) and washed five times with 700 µl PBS, before loading 300 µl trypsinized sample and incubation at 25 °C, 1000 rpm for 1 h. Unbound peptides were collected, immediately stabilized with 1% Trifluoroacetic acid and stored frozen until MS analysis. The avidin resin was washed five times with 600 µl bicarbonate, before eluting the R-SOX peptides with 300 µl 10 mM DTT at 37 °C, 2000 rpm for 2 h, which were collected and stabilized with 1% TFA. Finally, after washing the R-SNO peptides were eluted with 300 µl of 7 M guanidine-HCl at 95 °C for 30 min.

### Mass spectrometry analysis

Mass spectrometry analysis was performed by using five biological replicate samples, all combined with a heavy isotope-labeled reference sample as described above. Thus, the four series of samples (aerobic biofilm, aerobic planktonic, anaerobic biofilm, anaerobic planktonic) were analyzed in independent LC-MS/MS runs. Prior to LC-MS/MS analysis, digested peptides were desalted on a C18 microcolumn (Zip-Tip, Millipore), eluted in 2 µl of 60% acetonitrile (ACN), 0.1% aqueous formic acid (FA) and added 18 µl of 0.1% FA. Each sample was concentrated (5 µl) on a C18 cartridge (Dionex Acclaim PepMap100, 5 µm, 300 µm i.d. × 5 mm) and eluted on a capillary reverse-phase column (C18 Dionex Acclaim PepMap100, 3 µm, 75 µm i.d. × 50 cm) at 220 nl/min, with a gradient of 2–50% of buffer B in 180 min; (buffer A: 0.1% aq. FA/ACN 98:2 (v/v); buffer B: 0.1% aq. FA/ACN 90:10 (v/v)), coupled to a quadrupole Orbitrap mass spectrometer (Q Exactive, Thermo Fisher Scientific) using a top 10 data-dependent acquisition MS experiment: 1 survey MS scan (400–2,000 m/z; resolution 70,000) followed by 10 MS/MS scans on the 10 most intense precursors (dynamic exclusion of 30 s, resolution 17,500).

### Data analysis

Database search using MaxQuant (version 1.3.0.5) on SwissProt database (2017_05) was performed with methionine oxidation, cysteine carbamidomethylation (IAM), and biotinylation (iodoacetyl-PEG2-biotin, IAM-biotin) as variable modifications, with the following conditions: first search error tolerance 20 ppm, main error tolerance 6 ppm, MS/MS error tolerance 20 ppm, FDR 1%. A fold change was calculated for each condition: internal standard/aerobic biofilm, internal standard/aerobic planktonic.

#### Quantitative profiles

Quantitative profiles were generated by reprocessing the data from MaxQuant evidence file. Protein expression profiles were estimated using cysteine-free peptides only. We considered peptides carrying only one cysteine. Cysteine-containing peptides were classified according to their oxidation state (IAM labeled for reduced, IAM-biotin for S-NO, unmodified for S-OX). When a given cysteine residue was detected in different peptide forms (miss cleavages or oxidized methionine containing peptides), the features were aggregated using the median value of the ratios. The fold changes of cysteine-containing peptide were normalized to the related protein fold changes in order to avoid quantification bias induced by the differential protein expression.

The randomness of the datasets was tested by the non-parametric Wald–Wolfowitz runs test (*p* value threshold 0.05) using the software SPSS 26 IBM (Supplementary Table S6). The null hypothesis tests were performed by comparing the modified peptide fold change to the associated protein fold change. The normalized ratios (cysteine fold changes and protein fold changes) with the corresponding calculated variances were used to perform Student’s test between biofilm and planktonic conditions. Adjusted *p* values < 0.05 (Benjamini–Hochberg FDR correction) and fold changes >1.75 were chosen as significant thresholds.

Proteins detected in four or five replicates and absent in the other conditions were labeled as “biofilm only” or “planktonic only”. The same procedure was used for the cysteine-containing peptides, considering only species associated to proteins detected in both conditions.

Proteins with significant fold changes were used to create a dataset of “upregulated” and “downregulated” entries. The datasets were uploaded in STRING^72^ to perform protein network analyses and gene ontology enrichments, using K-means clustering, which produced six clusters.

### Reporting summary

Further information on research design is available in the Nature Research Reporting Summary linked to this article.

## Supplementary information

Supplementary Information

Reporting Summary

Supplementary Data 1

Supplementary Data 2

Supplementary Data 3

## Data Availability

Data are available via ProteomeXchange under the project Name: “Redox proteomics analysis of *E. coli* biofilms” with the following accession number: PXD020249.
