# Supplementary material for: Lifestyle-specific S-nitrosylation of protein cysteine thiols regulates Escherichia coli biofilm formation and resistance to oxidative stress
Source: NPJ Biofilms Microbiomes. 2021 Apr 13;7:34. doi: 10.1038/s41522-021-00203-w (PMC8044216; doi:10.1038/s41522-021-00203-w)
Supplement: Supplementary file 1 — Supplementary Information [file 41522_2021_203_MOESM1_ESM.pdf]

SUPPLEMENTARY INFORMATION

SUPPLEMENTARY FIGURES (supplementary Figures S1-S9)

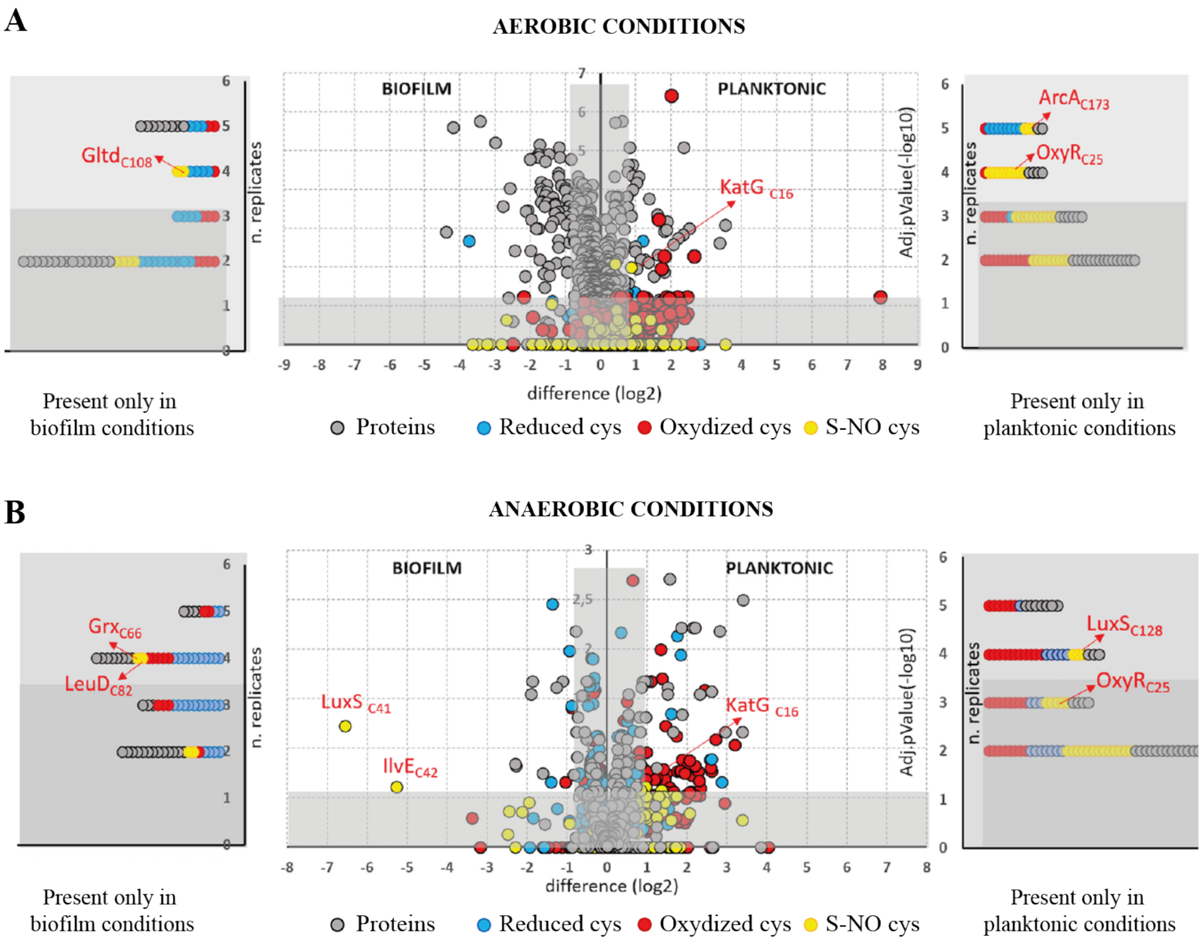

Supplementary Figure S1. Volcano plots combining the global dataset of the cysteine redox proteomics analysis of aerobic (panel A) and anaerobic (panel B) *E. coli* biofilm and planktonic cultures. Proteins appear in grey, reduced cysteines in blue, reversibly oxidized S-OX cysteines in red, *S*-nitrosylated S-NO cysteines in yellow. The lateral panels show the features detected specifically to one lifestyle. The dark grey areas delimit the non-significant data (thresholds as described in the method section).

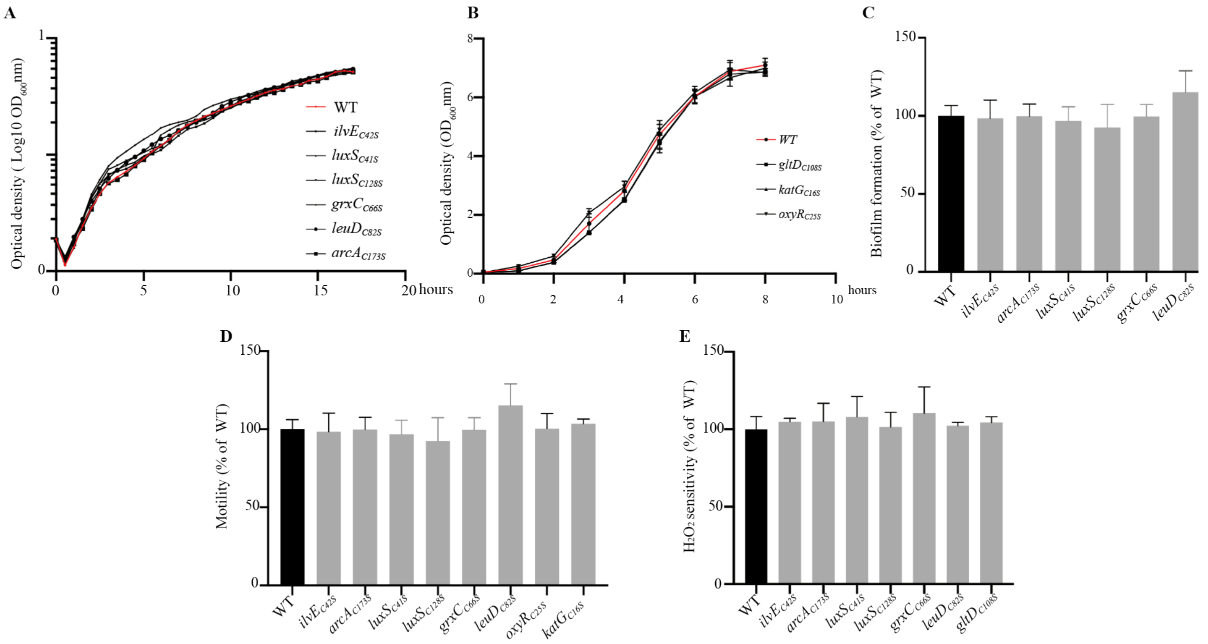

Supplementary Figure S2. Phenotypic analysis of mutants in genes corresponding to proteins with differentially *S-nitrosylated* cysteines. A: Growth kinetics were assessed in aerobic planktonic conditions in LB medium for *E. coli* WT (MG1655 F'*tet*), *grxC*$_{C66S}$, *leuD*$_{C82S}$, *ilvE*$_{C42S}$, *luxS*$_{C41S}$, *luxS*$_{C128S}$, *arcA*$_{C173S}$ mutants. **B**: Growth kinetics were assessed in aerobic planktonic conditions in LB medium for *oxyR*$_{C25S}$, *katG*$_{C16S}$ and *gltD*$_{C108S}$ mutants and WT strain. **C**: Biofilm biomass formation in microfermentors after 24 h in LB medium for *grxC*$_{C66S}$, *leuD*$_{C82S}$, *ilvE*$_{C42S}$, *luxS*$_{C41S}$, *luxS*$_{C128S}$, *arcA*$_{C173S}$ mutants and WT strains. The level of biofilm formed by the WT strain was set to 100%. **D**: Motility assays on 0.3% agar plates, incubated overnight at 30°C for *grxC*$_{C66S}$, *leuD*$_{C82S}$, *ilvE*$_{C42S}$, *luxS*$_{C41S}$, *luxS*$_{C128S}$, *arcA*$_{C173S}$, *oxyR*$_{C25S}$, *katG*$_{C16S}$ mutants and WT strains. The diameter of the growth zone was measured and set to 100% for the WT strain. **E**: Sensitivity to H_2O_2 oxidative stress for *grxC*$_{C66S}$, *leuD*$_{C82S}$, *ilvE*$_{C42S}$, *luxS*$_{C41S}$, *luxS*$_{C128S}$, *arcA*$_{C173S}$ and *gltD*$_{C108S}$ mutants and WT strains. The distance from the edge of the disk to the edge of the growth zone was measured and was set to 100% for the WT strain. All experiments were performed in triplicate, mean values are reported and error bars represent standard deviations.

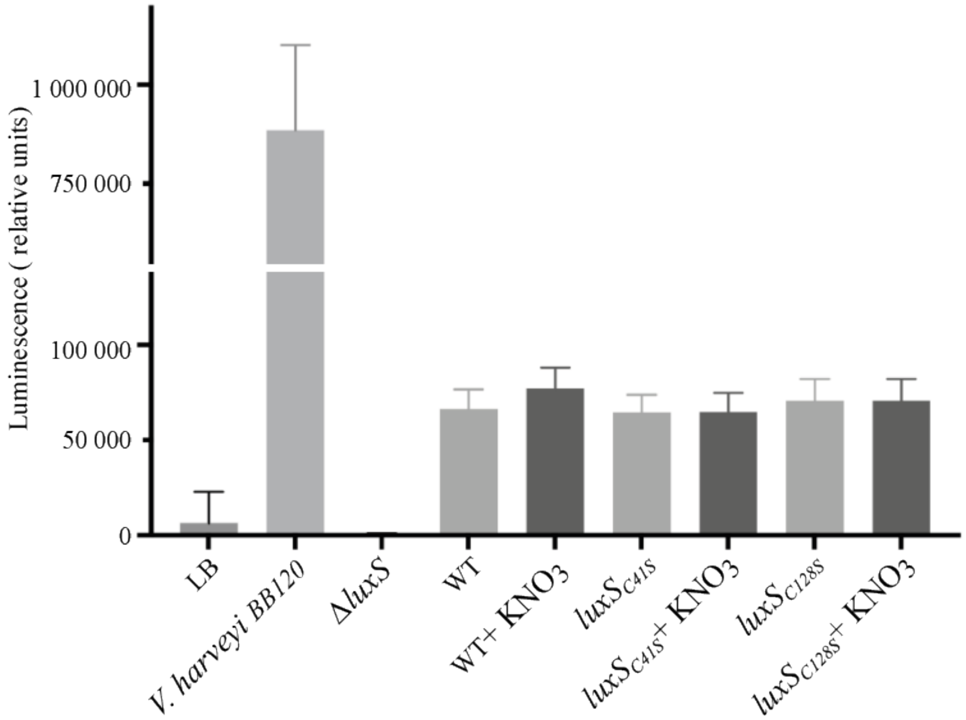

Supplementary Figure S3. Detection of AI-2 production by measuring the level of bioluminescence induced in the *V. harveyi* BB170 reporter strain (see Materials and Methods). *V. harveyi* BB120 grown in AB medium was used as a positive control and LB medium or MG1655Δ*luxS* as negative control.
Cell-free supernatants from WT, *luxS*$_{C41S}$ and *luxS*$_{C128S}$ strains were grown at 30°C in LB, in presence or absence of 10 mM KNO_3 and were tested for AI-2 activity. Luminescence level is expressed as luminescence relative units. Data represent the means and standard errors of the means of the results from three independent experiments.

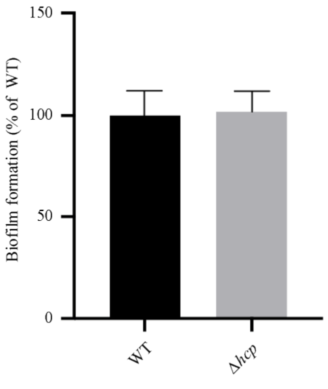

Supplementary Figure S4. Δ*hcp* mutant **biofilm formation.** Biofilms were cultivated in microfermentors in LB medium for 24 h. The level of biofilm formed by the *E. coli* WT (MG1655 F'*tet*) strain was set to 100%. Data are the means from three independent experiments.

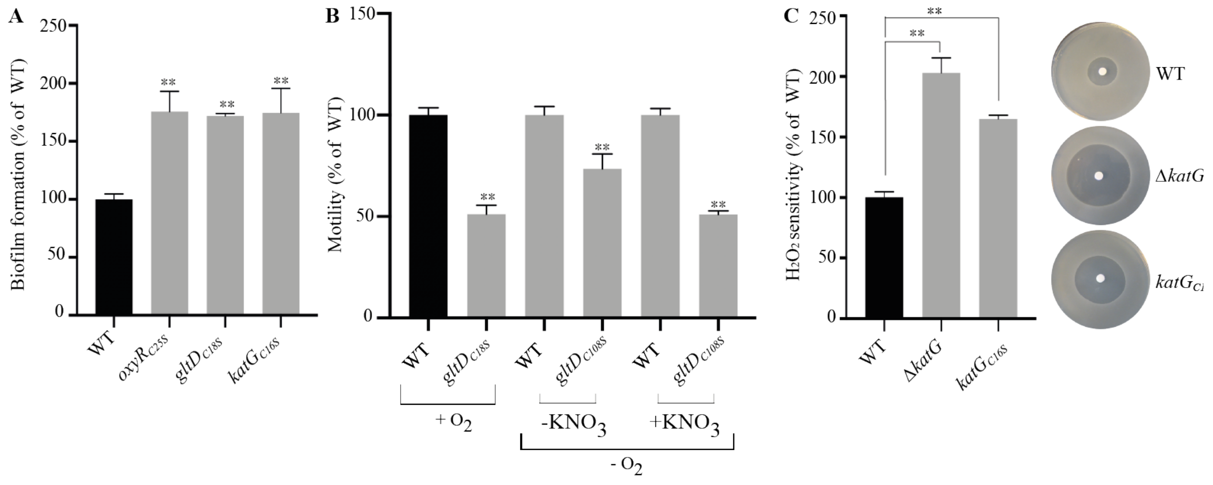

Supplementary Figure S5 Phenotypes associated with OxyR, KatG and GltD *S*-nitrosylation in M63B1 minimal medium. A: Biofilms of *E. coli* WT, *oxyR*$_{C25S}$, *katG*$_{C16S}$ and *gltD*$_{C108S}$ mutants, were grown in continuous-flow microfermentors for 48 h in M63B1 glucose medium before quantifying the biofilm biomass. The level of biofilm formed by the WT strain was set to 100%. Data are the means from three experiments. **B**: The motility of *E. coli* WT (MG1655 F'*tet*) and *gltD*$_{C108S}$ mutant were compared in M63B1 glucose medium in aerobic conditions and in anaerobic conditions in presence of KNO_3. Assays were performed on 0.3% agar plates and incubated overnight at 30°C. The diameter of the growth zone was measured and set to 100% for each WT strain. **C**: Sensitivity to H_2O_2 oxidative stress of a *katG*$_{C16S}$ mutant compared to the WT. The distance of growth inhibition from the edge of the disk to the edge of the growth zone was measured and was set to 100% for the WT strain. All experiments were performed in triplicate, mean values are reported and error bars represent standard deviations. ** $p \leq 0.05$, *** $p \leq 0.01$.

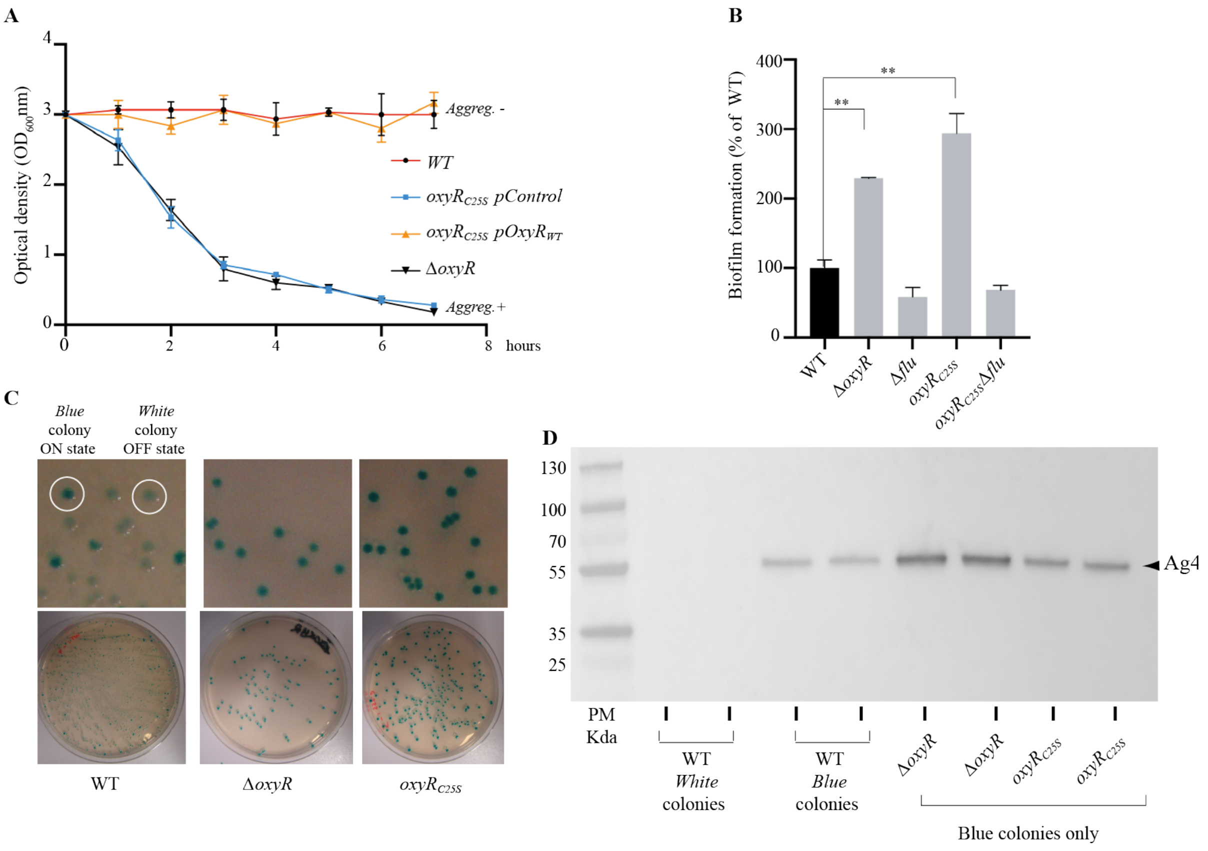

Supplementary Figure S6. **Impairing *S*-nitrosylation status of OxyR-Cys25 affects aggregation and *flu*-dependent biofilm formation A**: Cell aggregation kinetics for *oxyR*$_{\text{C25S}}$ mutant, complemented with corresponding plasmid-based allele or empty vector. **B**: Biofilm formation of *oxyR*$_{\text{C25S}}$ and Δ*flu* mutants. The level of biofilm formed by the WT strain was set to 100%. All experiments were performed in triplicate, mean values are reported and error bars represent standard deviations. ** $p \leq 0.05$, *** $p \leq 0.01$. **C**: *flu*(=*ag43*)-*lacZ* transcriptional fusions in a WT, Δ*oxyR* and *oxyR*$_{\text{C25S}}$ background plated on LB agar + X-gal plates: Blue colony: Ag43 expression in an ON state. White colony: Ag43 expression in an OFF state. **D:** Immunodetection of Ag43 in biofilm cultures of 2 white or blue colonies of WT, 2 blue colonies of Δ*oxyR* and 2 blue colonies of *oxyR*$_{\text{C25S}}$ using an anti-Ag43 polyclonal antibody.

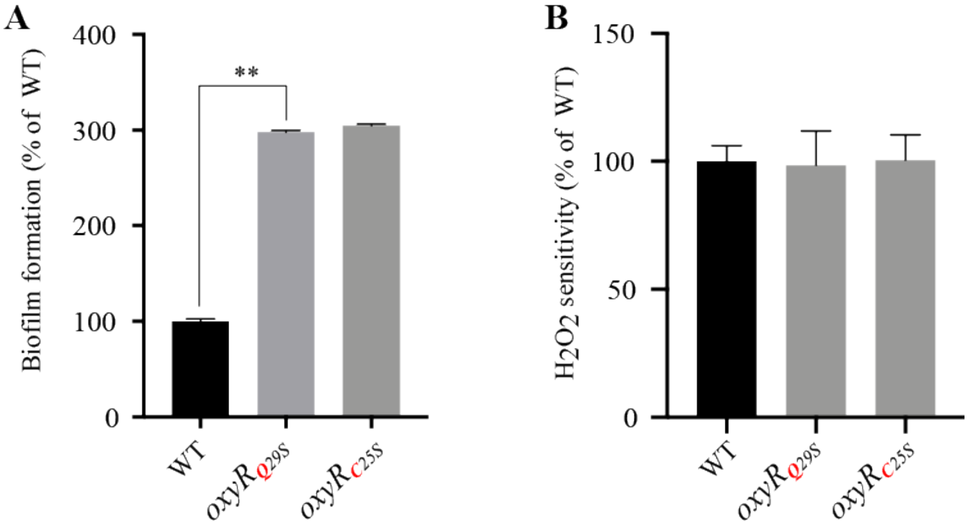

Supplementary Figure S7. Impairing OxyR DNA binding site by mutating increases biofilm formation but has no impact on *E. coli* H_2O_2 sensitivity

A: Biofilms of *E. coli* WT, *oxyR*$_{C25S}$ and *oxyR*$_{Q29S}$ mutants were grown in continuous flow microfermentors for 24 h in LB medium before quantifying the biofilm biomass. The level of biofilm formed by the WT strain was set to 100%. B: Sensitivity to H_2O_2 oxidative stress of *oxyR*$_{C25S}$ and *oxyR*$_{Q29S}$ mutants compared to the WT. The distance of growth inhibition from the edge of the disk to the edge of the growth zone was measured and was set to 100% for the WT strain. All experiments were performed in triplicate, mean values are reported and error bars represent standard deviations. ** $P \leq 0.05$, *** $P \leq 0.01$.

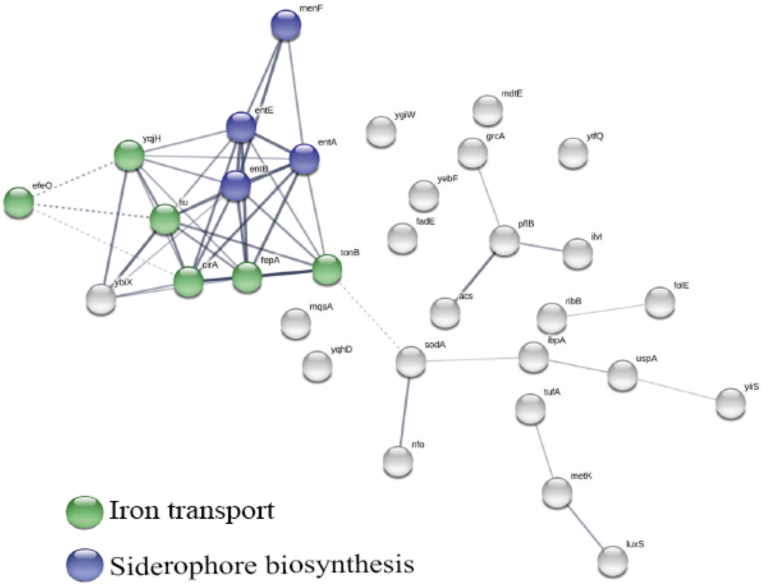

Supplementary Figure S8: STRING network analysis of proteins downregulated in biofilms. One significative cluster was obtained by k-means clustering. The proteins belonging to the cluster are involved in siderophore biosynthesis (blue spheres) and iron transport (green spheres).

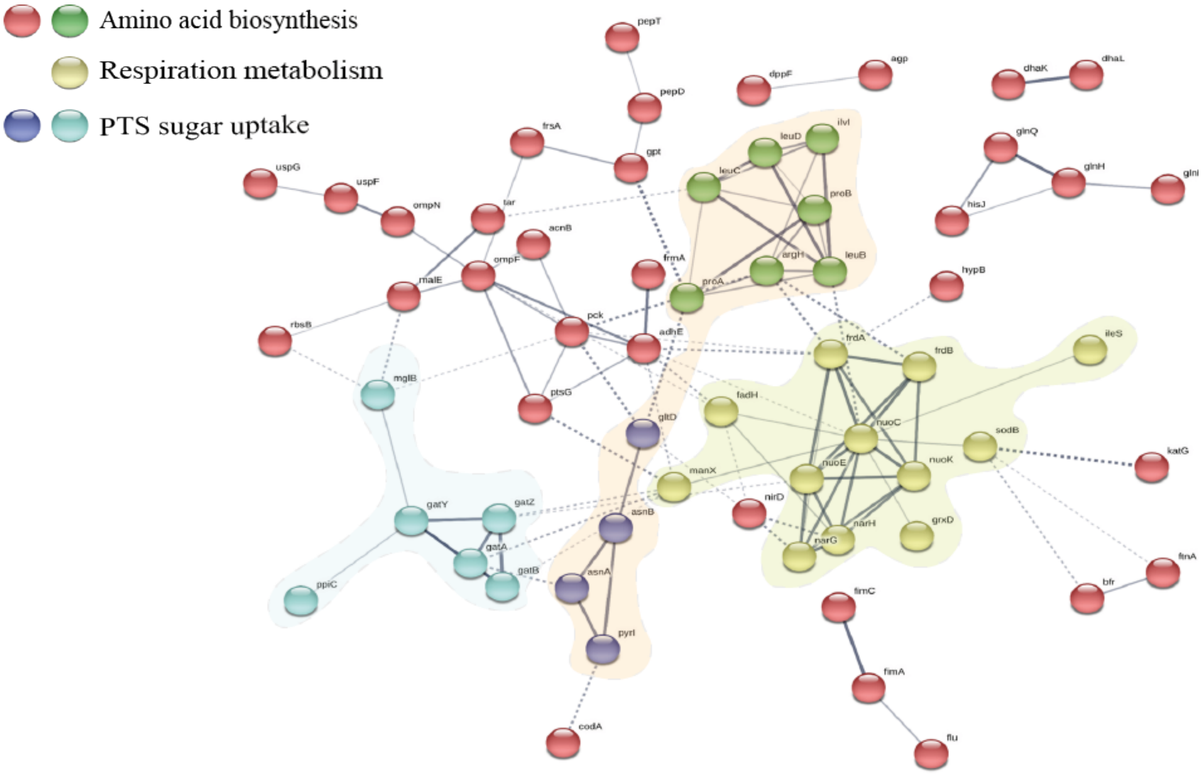

Supplementary Figure S9: STRING network analysis of proteins upregulated in biofilms. Three significative clusters were obtained by k-means clustering. Proteins in the cluster represented in yellow are involved in the respiratory chain. It is characterized by 11 oxidoreductase proteins, 7 of which (NuoC, NuoE, NuoK, NarG, NarH, FrdA, FrdB) are involved in respiration. Protein clusters represented in green and dark blue are involved in amino acid biosynthesis (LeuA, LeuC, LeuD, ProA, ProB, IlvI, ArgH) and (AsnA, AsnB, GltD, PyrL). Proteins in the light blue cluster belong to the PEP group translocation mechanism for sugar uptake and consists of 6 proteins, 4 of which (GatA, GatB, GatY, GatZ) belong to a PTS system involved in galactitol uptake.

SUPPLEMENTARY TABLES (supplementary Tables S1-S5).

Supplementary Table S1. List of peptides differentially reduced, *S*-oxidized and *S*-nitrosylated in biofilm *vs* planktonic *E. coli* cultures under aerobic conditions, by using the biotin-switch SILAC method. PO, planktonic with O_2; BO, biofilm with O_2. Content description in the first sheet of the Excel spreadsheet.

Supplementary Table S2. List of peptides differentially reduced, *S*-oxidized and *S*-nitrosylated in biofilm *vs* planktonic *E. coli* cultures under anaerobic conditions, by using the biotin-switch SILAC method. PN, planktonic without O_2; BN, biofilm without O_2. Content description in the first sheet of the Excel spreadsheet.

Supplementary Table S3. List of proteins identified to be differentially expressed in biofilm *vs* planktonic *E. coli* cultures by using the biotin-switch SILAC method. PO, planktonic with O_2; PN, planktonic without O_2; BO, biofilm with O_2; BN, biofilm without O_2. Content description in the first sheet of the Excel spreadsheet.

Supplementary table S4. Bacterial strains and plasmids used in this study

**Bacterial strain**	**Genotype**	**Reference**
E. coli **strains**		
MG1655 F'*tet* (=WT)	*E. coli* K-12 F$^+$ Δ*traD*; Tet^R, biofilm-forming strain	(Ghigo, 2001)
MG1655Δ*lacIZ-endflu-k*	Δ*lacIZ::KmFRT endflu-lacZ::Zeo.* Km^R, Zeo^R	Laboratory collection
MG1655Δ*lacIZ- endflu-c*	Δ*lacIZ::Cm* with *flu-lacZ* fusion. Cm^R, Zeo^R	(Chauhan et al., 201
MG1655 Δ*luxS*	Δ*luxS::Cm*, Cm^R	Laboratory collection
MG1655 Δ*lysA*Δ*argA*	Δ*lysA*::FRT and Δ*argA*::KmFRT in MG1655; Km^R	This study
MG1655F'Δ*lacIZ::Cm*	Deletion of *lacIZ* in MG1655F'tet. Cm^R	Laboratory collection
MG1655 F'*tet* Δ*lysA*Δ*argA*	Δ*lysA*::FRT and Δ*argA*::KmFRT in MG1655 F'*tet;*Tet^R, Km^R	This study
arcA$_{C173S}$	MG1655 F'*tet* with single nucleotide mutation in *arcA*-C173S	This study
gltD$_{C108S}$	MG1655 F'*tet* with single nucleotide mutation in *gltD*-C108S	This study
grxC$_{C66S}$	MG1655 F'*tet* with single nucleotide mutation in *grxC*-C66S	This study
ilvE$_{C42S}$	MG1655 F'*tet* with single nucleotide mutation in *ilvE*-C42S	This study
katG$_{C16S}$	MG1655 F'*tet* with single nucleotide mutation in *katG*-C16S	This study
leuD$_{C82S}$	MG1655 F'*tet* with single nucleotide mutation in *leuD*-C82S	This study
luxS$_{C41S}$	MG1655 F'*tet* with single nucleotide mutation in *luxS*-C41S	This study
luxS$_{C128S}$	MG1655 F'*tet* with single nucleotide mutation in *luxS*-C128S	This study
oxyR$_{C25S}$	MG1655 F'*tet* with single nucleotide mutation in *oxyR*-C25S	This study
oxyR$_{Q29S}$	MG1655 F'*tet* with single nucleotide mutation in *oxyR*-Q29S	This study
Δ*oxyR::Cm*	Deletion of *oxyR* in MG1655 F'*tet*, Cm^R	(Beloin et al., 2006)
Δ*oxyR::Km*	Deletion of *oxyR* in MG1655F'tet. Km^R	Laboratory collection
Δ*katG*	Deletion of *katG* in MG1655 F'*tet* Km^R	This study
Δ*flu*	Deletion of *flu* (=*ag43*) in MG1655 F'*tet*, Cm^R	(Chauhan et al., 2013)
Δ*hcp*	Deletion of *hcp* in MG1655 F'*tet*, Km^R	This study
oxyR$_{C25S}$Δ*flu*	Deletion of *flu* (=*ag43*) in *oxyR*$_{C25S}$, Cm^R	This study
Vibrio harveyi **strains**		
BB120	AI-1$^+$, AI-2$^+$	Laboratory collection
BB170	sensor 1$^-$, sensor 2$^+$	Laboratory collection
**Plasmids**		
pControl	Empty vector, pZS12, Amp^R	(Guet *et al.*, 2008)
pOxyR$_{WT}$	pACYC184 carrying WT *oxyR*, Cm^R	Laboratory collection
pOxyR$_{c25s}$	pACYC184 carrying *oxyR*$_{c25s}$ *allele*, Cm^R	This study
pKatG$_{WT}$	pZS12 carrying WT *katG*, Amp^R	This study
pGltD$_{WT}$	pZS12 carrying WT *gltD,* Amp^R	This study

Ghigo, J.M. (2001). Natural conjugative plasmids induce bacterial biofilm development. Nature *412*, 442-445.

Beloin, C., Michaelis, K., Lindner, K., Landini, P., Hacker, J., Ghigo, J.M., and Dobrindt, U. (2006). The transcriptional antiterminator RfaH represses biofilm formation in Escherichia coli. Journal of bacteriology *188*, 1316-1331.

Chauhan, A., Sakamoto, C., Ghigo, J.M., and Beloin, C. (2013). Did I pick the right colony? Pitfalls in the study of regulation of the phase variable antigen 43 adhesin. PloS one *8*, e73568.

C. C Guet, L. Bruneaux, T. L Min, D. Siegal-Gaskins, I. Figueroa, T. Emonet and P.Cluzel (2008). Minimally invasive determination of mRNA concentration in single living bacteria.Nucleic Acids Res . 2008 Jul;36(12):e73. doi: 10.1093/nar/gkn329.

Supplementary Table S5: List of primers used in this study

**Gene deletion mutants constructed by P1*vir* transduction from mutant strains of the Keio collection**	
Double auxotroph mutant constructions for SILAC experiments	
lysA-500-5	GCTGACATCGGGATAACGTGC
lysA-500-3	GCTCTAACCACTCTATTTCTG
argA-500-5	GCCGCCATCCCCTTGAGTACC
argA-500-3	GCGCAAAATTCATCGTCATCC
**MAGE construction of single nucleotide mutants in *E. coli***	
Primers with 5’ end phosphorothioation designed with MODEST online tool	
oxyR-C25S*	C*A*G*C*TCATCTTCCAGCTTACGAATTTGCCCGCTAAGCGTCGGCTGG CTAACGTGGgAGGAATCTGCCGCACGCCGAAAATGGCGGTGTTC
luxS-C41S*	A*G*C*A*AACAGGTGCTCCAGGGTATGGATCCCTCTTTCTGGCATCACT TCTTTGTTCGGCACGgAGAAGCGCAGATCGAACACGGTGATTGC
luxS-C128S*	G*A*A*T*GCTACGCGCAATATCCTGCGCTTCCTGCAACGAGTGCATCTG GTAAGTGCCAgACTGGTAGACGTTCAGTTCCGGGATCTGATTCT
grxC-C66S*	T*A*A*T*GCCAACGTTCACACGTTATTTCAGCAGGGGATCCAGTCCACC ACGTGCATCCAATGCATACAAGTCATCAgAGCCGCCAATGTGCT
ilvE-C42S*	G*C*G*G*AATACAACCGGTCCTTTGTGCGAGTCGTAGgAACGGATGCCT TCAAAAACCGAAGTGCCATAGTGCAGCGCGTGCGACATCACATG
arcA-C173S*	C*G*C*G*CCATGCTTCACTTCTcTGAAAACCCAGGCAAAATTCAGTCCC GTGCTGAACTGCTGAAGAAAATGACCGGCCGTGAGCTGAAACCG
gltD-C108S*	A*A*G*A*CCGTCTGTGCGAAGGTTCCTCCACTCTGAACGATGAGTTTGG CGCGGTGACCATCGGCAACATTGAGCGCTATATCAACGATAAAG
leuD-C82S*	A*A*A*C*CCGGACTTCGTGCTGAACTTCCCGCAGTATCAGGGCGCTTCC ATTTTGCTGGCACGAGAAAACTTCGGCTCTGGCTCTTCGCGTGA
katG-C16S*	T*A*A*C*ACTGTAGAGGGGAGCACATTGATGAGCACGTCAGACGATAT CCATAACACCACAGCCACTGGCAAA TCCCCGTTCCATCAGGGCGG
oxyR-Q29S*	C*T*G*G*TCCGCTCCAGCAACATCACGCCCAGCTCATCTTCCAGCTTAC GAATTTGCCCGCTAAGCGTCGGCgaGCTAACGTGGCAGGAATCT
Screening with HiDi polymerase	
oxyR-C25m-5	CGTGCGGCAGATTCCTC
oxyR-C25wt-5	CGTGCGGCAGATTCCTG
oxyR-C25-3	CTTGCCATCTCTTTAAGGAC
luxS-C41m-5	CGTGTTCGATCTGCGCTTCTC
luxS-C41wt-5	CGTGTTCGATCTGCGCTTCTG
luxS-C128m-3	CGAGTGCATCTGGTAAGTGCCAG
luxS-C128wt-3	CGAGTGCATCTGGTAAGTGCCAC
grxC-C66-5	GCCAATGTTGAAATC
grxC-C66m-3	CCAATGCATACAAGTCATCAG
grxC-C66wt-3	CCAATGCATACAAGTCATCAC
ilvE-C42m-5	GGTTTTTGAAGGCATCCGTTC
ilvE-C42wt-5	GGTTTTTGAAGGCATCCGTTG
ilvE-C42-3	ATCACCGACGAAGATCAG
gltD-C108wt-5	CGTCTGTGCGAAGGTTCCTG
gltD-C108m-5	CGTCTGTGCGAAGGTTCCTC
gltD-C108-3	CGTTACGCGTCAGGACATCC
leuD-C82wt-5	GCACGAGAAAACTTCGGCTG
leuD-C82m-5	GCACGAGAAAACTTCGGCTC
leuD-C82-3	CGAAATGGATCCCCGGATTAG
katG-C16wt-5	CACCACAGCCACTGGCAAATG

katG-C16m-5	CACCACAGCCACTGGCAAATC
katG-C16-3	CACGGTTGAGATTCTGTCAAC
oxyR-Q29wt-5	GATTCCTGCCACGTTAGCCA
oxyR-Q29m-5	GATTCCTGCCACGTTAGCTC
oxyR-Q29-3	CTTCATGCAGATACATTTCCAGC
arcA-C173-5	CTACATCACCAAACCGTTC
arcA-C173m-3	ATTTTGCCTGGGTTTTCAG
arcA-C173wt-3	ATTTTGCCTGGGTTTTCAC
Verification by sequencing	
oxyR-C25-seq-5	TCGTGATCTTGAGTACCTGGT
oxyR-C25-seq-3	CTGATCCACCAGCAGCATTC
luxS-C41-seq-5	GTTCGGGTGGCGAAAACAAT
luxS-C41-seq-3	CTTCCATTGCCGCTTTCCAG
luxS-C128-seq-5	CCAGATGAGCAGCGTGTTG
luxS-C128-seq-3	AACTTCTCTTTCGGCAGTGC
grxC-C66-seq-5	AAGAAACCTGCCCGTATTGC
grxC-C66-seq-3	GATCCAGTCCACCACGTG
ilvE-C42-seq-5	TGGTTCAATGGGGAGATGGT
ilvE-C42-seq-3	GCTCATCAATGCTCTGCGAA
gltD-C108-seq-5	GAGTCAGAATGTTTATCAATTTATC
gltD-C108-seq-3	CCCATGCCGGTGAAGATTTC
leuD-C82-seq-5	GGCAGAGAAATTTATCAAAC
leuD-C82-seq-3	CATAAACGCAGGTTGTTTTG
katG-C16-seq-5	GAGCACAAAATGCTGCCTCG
katG-C16-seq-3	CTTACGTTATCCGGCCAGG
arcA-C173-seq-5	TGGTTGGGAACTGGACATCA
arcA-C173-seq-3	CGTGAATGGTGGCGATGATT
**Gibson cloning primers for complementation experiments**	
katG-pZS12-REV	CTAATTAAGCTTTTACAGCAGGTCGAAACGGTCGAG
katG-pZS12-FOR	GAGAAAGGTACCATGAGCACGTCAGACGATATCCATA
gltD-pZS12-FOR	GAGAAAGGTACCATGAGTCAGAATGTTTATCAATTTA
gltD-pZS12-REV	CTAATTAAGCTTTTAAACTTCCAGCCAGTTCATAAT
pZS12-katG-REV	TGACGTGCTCATGGTACCTTTCTCCTCTTTAATGAAT
pZS12-katG-FOR	CTGCTGTAAAAGCTTAATTAGCTGAGTCTAGAGGC
pZS12-gltD-REV	AACATTCTGACTCATGGTACCTTTCTCCTCTTTAATGAAT
pZS12-gltD-FOR	GAAGTTTAAAAGCTTAATTAGCTGAGTCTAGAGGC

Supplementary Table S6. Test of the randomness of the datasets. Wald-Wolfowitz runs tests were performed to evaluate the non-randomness of the datasets. P-values are in the raw named "Asymp.Sig. (2-tailed)". All the run tests show p-value>0.05, meaning that the datasets are random.

Aerobic dataset

Runs Test

	Rox BO	Rred BO	Rsno BO	R BO	Rox PO	Rred PO	Rsno PO	R PO
Test Value[a]	.28	-.18	3.30	-.09	-.09	-.05	2.82	.03
Cases < Test Value	270	260	105	361	272	242	165	356
Cases >= Test Value	276	260	107	370	274	253	166	360
Total Cases	546	520	212	731	546	495	331	716
Number of Runs	286	280	105	378	286	266	160	372
Z	1.031	1.668	-.274	.856	1.028	1.587	-.715	.973
Asymp. Sig. (2-tailed)	.303	.095	.784	.392	.304	.113	.474	.330

a. Median

Anaerobic dataset

Runs Test

	Rox BN	Rred BN	Rsno BN	R BN	Rox PN	Rred PN	Rsno PN	R PN
Test Value[a]	.2207014165	-.167932037	3.264040811	-.063299200	-.302301504	-.058171905	2.681158000	-.001583608
Cases < Test Value	240	258	104	315	275	248	124	355
Cases >= Test Value	241	258	104	316	275	248	124	355
Total Cases	481	516	208	631	550	496	248	710
Number of Runs	231	267	116	311	280	257	127	345
Z	-.958	.705	1.529	-.438	.341	.719	.255	-.826
Asymp. Sig. (2-tailed)	.338	.481	.126	.661	.733	.472	.799	.409

a. Median
