# Supplementary material for: Lifestyle-specific S-nitrosylation of protein cysteine thiols regulates Escherichia coli biofilm formation and resistance to oxidative stress
Source: NPJ Biofilms Microbiomes. 2021 Apr 13;7:34. doi: 10.1038/s41522-021-00203-w (PMC8044216; doi:10.1038/s41522-021-00203-w)
Supplement: Supplementary file 2 — Reporting Summary [file 41522_2021_203_MOESM2_ESM.pdf]

nature research

Corresponding author(s): Jean-Marc GHIGO

Last updated by author(s): 01/21/2021

# Reporting Summary

Nature Research wishes to improve the reproducibility of the work that we publish. This form provides structure for consistency and transparency in reporting. For further information on Nature Research policies, see our Editorial Policies and the Editorial Policy Checklist.

## Statistics

For all statistical analyses, confirm that the following items are present in the figure legend, table legend, main text, or Methods section.

| n/a | Confirmed | |
|---|---|---|
| ☐ | ☒ | The exact sample size (*n*) for each experimental group/condition, given as a discrete number and unit of measurement |
| ☐ | ☒ | A statement on whether measurements were taken from distinct samples or whether the same sample was measured repeatedly |
| ☐ | ☒ | The statistical test(s) used AND whether they are one- or two-sided<br>*Only common tests should be described solely by name; describe more complex techniques in the Methods section.* |
| ☒ | ☐ | A description of all covariates tested |
| ☐ | ☒ | A description of any assumptions or corrections, such as tests of normality and adjustment for multiple comparisons |
| ☐ | ☒ | A full description of the statistical parameters including central tendency (e.g. means) or other basic estimates (e.g. regression coefficient) AND variation (e.g. standard deviation) or associated estimates of uncertainty (e.g. confidence intervals) |
| ☐ | ☒ | For null hypothesis testing, the test statistic (e.g. *F*, *t*, *r*) with confidence intervals, effect sizes, degrees of freedom and *P* value noted<br>*Give P values as exact values whenever suitable.* |
| ☒ | ☐ | For Bayesian analysis, information on the choice of priors and Markov chain Monte Carlo settings |
| ☐ | ☒ | For hierarchical and complex designs, identification of the appropriate level for tests and full reporting of outcomes |
| ☒ | ☐ | Estimates of effect sizes (e.g. Cohen's *d*, Pearson's *r*), indicating how they were calculated |

*Our web collection on statistics for biologists contains articles on many of the points above.*

## Software and code

Policy information about availability of computer code

Data collection — *Provide a description of all commercial, open source and custom code used to collect the data in this study, specifying the version used OR state that no software was used.*

Data analysis — *Provide a description of all commercial, open source and custom code used to analyse the data in this study, specifying the version used OR state that no software was used.*

For manuscripts utilizing custom algorithms or software that are central to the research but not yet described in published literature, software must be made available to editors and reviewers. We strongly encourage code deposition in a community repository (e.g. GitHub). See the Nature Research guidelines for submitting code & software for further information.

## Data

Policy information about availability of data

All manuscripts must include a data availability statement. This statement should provide the following information, where applicable:

- Accession codes, unique identifiers, or web links for publicly available datasets
- A list of figures that have associated raw data
- A description of any restrictions on data availability

Data are available via ProteomeXchange with identifier PXD020249.



April 2020

# Field-specific reporting

Please select the one below that is the best fit for your research. If you are not sure, read the appropriate sections before making your selection.

☒ Life sciences ☐ Behavioural & social sciences ☐ Ecological, evolutionary & environmental sciences

For a reference copy of the document with all sections, see nature.com/documents/nr-reporting-summary-flat.pdf

# Life sciences study design

All studies must disclose on these points even when the disclosure is negative.

| | |
|---|---|
| Sample size | We calculated the number of necessary replicates according to the equation 12 from the paper "Statistical design of quantitative mass spectrometry-based proteomic experiment" (J Proteome Res. 2009 May;8(5):2144-56. doi: 10.1021/pr8010099). It results that 4 biological replicates are necessary to distinguish 1.7 fold changes with aType I error of 5%, a Type II error of 80% and with the variances associated to our experimental conditions. However, we have chosen to use 5 biological replicates to obtain more robust results. |
| Data exclusions | no data were excluded |
| Replication | biological triplicate led to reproducible results as indicated by mean values are reported and error bars representing standard deviationsest |
| Randomization | Samples were randomly analyzed by LC-MSMS. Our experimental design does not allow to randomize the SILAC labeling. This because the heavy labeling is used to generate an internal stadard sample. Moreover, biofilm bacteries do not have the same rate of isotope labeling than planktonic cells. |
| Blinding | The investigators were not blinded to during experiments and outcome assessment. |

# Reporting for specific materials, systems and methods

We require information from authors about some types of materials, experimental systems and methods used in many studies. Here, indicate whether each material, system or method listed is relevant to your study. If you are not sure if a list item applies to your research, read the appropriate section before selecting a response.

## Materials & experimental systems

| n/a | Involved in the study |
|---|---|
| ☒ | ☐ Antibodies |
| ☒ | ☐ Eukaryotic cell lines |
| ☒ | ☐ Palaeontology and archaeology |
| ☒ | ☐ Animals and other organisms |
| ☒ | ☐ Human research participants |
| ☒ | ☐ Clinical data |
| ☒ | ☐ Dual use research of concern |

## Methods

| n/a | Involved in the study |
|---|---|
| ☒ | ☐ ChIP-seq |
| ☒ | ☐ Flow cytometry |
| ☒ | ☐ MRI-based neuroimaging |
